# Feature Extraction of the Brain’s Dynamic Complex Network Based on EEG and a Framework for Discrimination of Pediatric Epilepsy

**DOI:** 10.3390/s22072553

**Published:** 2022-03-26

**Authors:** Zichao Liang, Siyang Chen, Jinxin Zhang

**Affiliations:** Department of Medical Statistics, School of Public Health, Sun Yat-sen University, Guangzhou 510080, China; liangzch3@mail2.sysu.edu.cn (Z.L.); chensy273@mail2.sysu.edu.cn (S.C.)

**Keywords:** dynamic complex network, feature extraction, sliding window analysis, EEG, pediatric epilepsy

## Abstract

Most of the current complex network studies about epilepsy used the electroencephalogram (EEG) to directly construct the static complex network for analysis and discarded the dynamic characteristics. This study constructed the dynamic complex network on EEG from pediatric epilepsy and pediatric control when they were asleep by the sliding window method. Dynamic features were extracted and incorporated into various machine learning classifiers to explore their classification performances. We compared these performances between the static and dynamic complex network. In the univariate analysis, the initially insignificant topological characteristics in the static complex network can be transformed to be significant in the dynamic complex network. Under most connectivity calculation methods between leads, the accuracy of using dynamic complex network features for discrimination was higher than that of static complex network features. Particularly in the imaginary part of the coherency function (iCOH) method under the full-frequency band, the discrimination accuracies of most machine learning classifiers were higher than 95%, and the discrimination accuracies in the higher-frequency band (beta-frequency band) and the full-frequency band were higher than that of the lower-frequency bands. Our proposed method and framework could efficiently summarize more time-varying features in the EEG and improve the accuracies of the discrimination of the machine learning classifiers more than using static complex network features.

## 1. Introduction

Epilepsy is a chronic non-communicable brain disease. According to WHO’s estimation, approximately 50 million people worldwide are affected [1]. It is also one of the most common neurological diseases in children, with an incidence of 33.3–82 cases per 100,000 per year [2]. The incidence of pediatric epilepsy (PE) is highest in the first year of life (infancy) and then gradually decreases [3]. The potential causes of seizures in children include fever, meningitis, metabolic imbalances, exposure to toxins, head injuries, tumors, or other uncertain triggers. The frequency of epileptic seizures ranges from less than once a year to several times a day. Additionally, the misdiagnosis rate of epilepsy in children is at least 25% [2]. Population-based studies have shown that after regular treatment, nearly two-thirds of epileptic children become seizure-free within 3 to 5 years [4], and almost half of the patients can successfully stop anti-epileptic drugs [5]. Therefore, timely diagnosis and treatment for children with epilepsy are crucial.

An electroencephalogram (EEG) captures the changes in the brain’s electric field to obtain the activities of the nervous system, which has significant reference value in clinical diagnosis and research. Clinicians widely use EEG in auxiliary diagnosis because of its non-invasive simple operation, low cost, and high time resolution characteristics. In diagnosis, EEG can be used to detect the possibility of increased risk of epilepsy, ongoing seizures, or areas of potential cerebral dysfunction [6]. After the patient is diagnosed, EEG can also assist in diagnosing epilepsy syndrome, determining whether to undergo surgery, choosing proper anti-epileptic drugs or time to stop them, assessing the compliance, and predicting the prognosis [7].

In clinical practice, EEG is usually observed with human eyes and assisted in diagnosis by identifying characteristics. However, this procedure inevitably contains subjectiveness. Doctors with different comprehensive abilities to EEG, artifacts in EEG caused during signals collection, or fatigue caused by the monotonous task of perpetual EEG reading, etc., may lead to errors in EEG manual reports, which consumes a lot of time and workforce. In addition, as the brain is a complex and non-linear dynamic system [8], it is challenging to discover the complex information contained in the EEG through human eyes’ discrimination.

In recent years, with the progress and development of brain science, more and more scholars have used graph theory and complex network theory supported by brain science to analyze the structure and function of the brain’s neural networks and define the relationship with brain activities through graph theory [9]. The vertexes and edges of the network are formed and quantified to provide a basis for discovering brain functions, structures, and abnormal activities’ characteristics, to have a deeper understanding of various diseases related to the brain and their underlying mechanism of changes. At the same time, with the support of machine learning theory, a variety of machine learning classifiers can be used to automatically distinguish and classify features found in complex networks without manual intervention [10,11,12]. In the research on the brain’s networks of patients with epilepsy, various studies have used complex network theory. It can distinguish between epilepsy patients’ (ictal phase, depth electrodes were implanted symmetrically into the hippocampal formations) EEG and healthy controls’ (HCs’) EEG using the visual graph (VG) method with an accuracy of more than 95% [13], which can also differentiate between epilepsy patients’ (interictal phase) and HCs’ EEG with an accuracy of more than 85% [14].

In previous studies on various brain diseases, there were few studies focused on the construction of dynamic complex networks using EEG [15,16]. Most studies have directly constructed the static complex brain network for analysis using a period of EEG time series, discarding brain networks’ time-varying dynamic characteristics. At the same time, the network construction methods and the dynamic feature extraction methods under each window are also different and need further research for comparison. This research used the functional connectivity calculation methods between EEG leads to construct the brain’s dynamic complex network by sliding window. Additionally, we calculated the topological features of the networks under each window to form multiple time series of characteristics, extracted the characteristics of each time series as the features of the dynamic brain complex network, and incorporated them into a variety of machine learning classifiers to explore their discrimination performance in the classification models.

## 2. Materials and Methods

### 2.1. Research Participants

From January 2019 to June 2021, 36 participants were enrolled in the Pediatric Department of Shenzhen People’s Hospital, and we collected their raw EEG data. This project was approved by the ethics committee of the School of Public Health in Sun Yat-sen University (2021-No.081) and obtained the research participants’ agreements. The participants included two groups: the first group (pediatric control, PC) consisted of 20 healthy children (7 females, 7.05 ± 3.53 years old). They came to the hospital with the symptoms of convulsions, abdominal pain, and limb shaking and underwent an EEG examination but were not diagnosed with any disease. The second group (PE) consisted of 16 patients (6 females, 7.75 ± 4.92 years old) diagnosed with epilepsy by clinicians.

The diagnosis of epilepsy was based on the International League Against Epilepsy (ILAE) [17]. The inclusion criteria were: (1) at least two unprovoked (or reflex) seizures occurring >24 h apart; (2) one unprovoked (or reflex) seizure and a probability of further seizures such as the general recurrence risk (at least 60%) after two unprovoked seizures; (3) diagnosis of an epilepsy syndrome. The exclusion criteria were: (1) other infectious diseases, cerebrovascular diseases, poisoning, or metabolic encephalopathy; (2) nervous system tumors, myelopathy, peripheral neuropathy; (3) primary mental disorders, brain trauma, brain tumors, or other neurological diseases; (4) have ever taken any anti-seizure medication.

All participants were homogeneous for gender and age. The hypothesis test did not show any statistically significant difference between the sex of the two groups (*χ*^2^ = 0.024, *p* = 0.877). In addition, the hypothesis test did not find any statistically significant difference in the average age between the two groups (*U* = 163.00, *p* = 0.923). See Table 1.

### 2.2. EEG Signals and Preprocessing

Each participant could perform routine long-term EEG recording. We used the Nicolet EEG machine to record the EEG at a sampling rate of 500 Hz. The scalp electrodes were placed under the international 10–20 montage system, and the A1 and A2 electrodes were used as references. EEG was performed in the same recording room using the same system, and the same EEG technician used conventional measurement techniques to determine the electrodeposition. We collected EEG for at least 15 h with the subjects relaxed, asleep, and their eyes closed to avoid disturbance. All EEG records contained 19 scalp electrodes (Fp1, Fp2, F7, F3, Fz, F4, F8, T3, C3, Cz, C4, T4, T5, P3, Pz, P4, T6, O1, and O2) and a visual inspection by EEG technician was performed.

We extracted the ictal phase data from raw EEG of PE group under the guidance of professional neurology clinicians; as a result, we had only various ictal phases linked together of each PE participant in the EEG data. To reduce data volume and speed up computation, we down-sampled the data to 100 Hz. Then, the data was filtered with band-pass at frequencies of 0.5 and 45 Hz. Finally, automated artifact removal was performed on the manually processed dataset using the independent components algorithm (ICA). These preprocessing steps were operated using EEGLab toolbox [18] in MATLAB (MathWorks).

### 2.3. The Construction of the Brain’s Static and Dynamic Complex Networks

#### 2.3.1. Constructing the Original EEG Dataset and the Split-Fragment EEG Dataset

Due to the relatively small number of participants included in this research (16 participants of the PE group and 20 participants of the PC group), while using the original EEG (each EEG signal included in the discriminate model came from a different participant) to construct the models, we divided some more prolonged original EEG into multiple segments (to obtain more EEG fragments and ensure that each fragment had enough length for sliding window analysis, the signal length of each segment we chose was at least 100 s). Finally, 61 segments in the PE group and 72 segments in the PC group were formed as the split-fragment EEG dataset. Model training and discrimination were performed separately on the two kinds of datasets mentioned above.

#### 2.3.2. Frequency Filtering

We used a band-pass filter to extract the signals in the specified frequency band and decomposed the pre-processed EEG signals into the following six frequency bands: Delta (0.5–4 Hz), Theta (4–8 Hz), Alpha-1 (8–10 Hz), Alpha-2 (10–12 Hz), Beta (12–30 Hz), and full-frequency band signals (0.5–45 Hz). In this step, we used the pop_eegfiltnew function of the EEGLab toolbox in MATLAB.

#### 2.3.3. The Calculation of the Connectivity and Connectivity Strength between EEG Leads

Assume that *X*(*t*) and *Y*(*t*) represent the EEG electrodes *X* and *Y*, respectively. We proposed using the following four methods to calculate the connectivity and connectivity strength between EEG leads:

A. Based on coherence: magnitude square coherence (MSC), imaginary part of the coherency function (iCOH).

a. MSC is used to find the dependency between two signals [19], the calculation is as follows:

The fast Fourier-transform method is used to convert the time domain signals *X*(*t*) and *Y*(*t*) to the frequency domain. Then, for each frequency *f*, its respective frequency power density *S_xx_*(*f*) and *S_yy_*(*f*) and their cross power spectral density *S_yy_*(*f*) are estimated. We used Equation (1) to calculate the coherence function *K_xy_*(*f*):

(1) $$K_{xy}\left(f\right)=\frac{S_{xy}\left(f\right)}{\sqrt{S_{xx}\left(f\right)S_{yy}\left(f\right)}}$$

Finally, Equation (2) is used to calculate the coherence value (MSC) at frequency *f*:

(2) $$MSC=COH_{xy}\left(f\right)={\left|K_{xy}\left(f\right)\right|}^{2}$$

The value range of MSC is 0~1, MSC =0 means that *X*(*t*) and *Y*(*t*) have no linear dependence on frequency *f*. The larger the coherence value, the stronger the statistical dependence between the two signals, and vice versa.

b. iCOH is the imaginary part of the coherence function *K_xy_*(*f*). It can not only find the dependence between the two signals but also avoid the influence caused by the volume conduction in the EEG signals [20].

B. Based on phase synchronization: phase lag index (PLI), which is used to measure the degree of phase synchronization between two signals, and it can also better exclude the influences of volume conduction in EEG signals [21]; the calculation is as follows:

Obtain the instantaneous phase time series of *X*(*t*) and *Y*(*t*) signals through Hilbert transform, *P.V.* is the Cauchy principal value:

(3) $$X_{H}\left(t\right)=\frac{1}{\pi }P.V.\int_{-\infty }^{+\infty }\frac{X\left(\tau \right)}{t-\tau }d\tau$$

(4) $$Y_{H}\left(t\right)=\frac{1}{\pi }P.V.\int_{-\infty }^{+\infty }\frac{Y\left(\tau \right)}{t-\tau }d\tau$$

Additionally, the analytical signals *X_an_*(*t*) and *Y_an_*(*t*) of *X*(*t*) and *Y*(*t*) can be obtained:

(5) $$X_{an}\left(t\right)=X\left(t\right)+iX_{H}\left(t\right)$$

(6) $$Y_{an}\left(t\right)=Y\left(t\right)+iY_{H}\left(t\right)$$

Using analytical signals, the instantaneous amplitude *A_x_*(*t*), *A_y_*(*t*) and instantaneous phase $\emptyset_{x}\left(t\right)$, $\emptyset_{y}\left(t\right)$, can be calculated:

(7) $$A_{x}\left(t\right)=\sqrt{X_{an}{\left(t\right)}^{2}+X_{H}{\left(t\right)}^{2}}$$

(8) $$A_{y}\left(t\right)=\sqrt{Y_{an}{\left(t\right)}^{2}+Y_{H}{\left(t\right)}^{2}}$$

(9) $$\emptyset_{x}\left(t\right)=tan^{-1}\frac{X_{H}\left(t\right)}{X_{an}\left(t\right)}$$

(10) $$\emptyset_{y}\left(t\right)=tan^{-1}\frac{Y_{H}\left(t\right)}{Y_{an}\left(t\right)}$$

We calculate Δ$\emptyset_{xy}\left(t\right)$, which is their phase difference at time *t*:

(11) $$\Delta \emptyset_{xy}\left(t\right)=|\emptyset_{x}\left(t\right)-\emptyset_{y}\left(t\right)|$$

In actual analysis, the phase difference needs to be converted to [0, 2π):

(12) $$\Delta \emptyset_{rel}\left(t\right)=\Delta \emptyset_{xy}\left(t\right)mod2\pi$$

The formula of PLI is:

(13) $$PLI=\left|\frac{1}{N}\overset{N}{\underset{n=1}{\sum }}sign\left(\Delta \emptyset_{rel}\left(t\right)\right)\right|$$

The value range of PLI is 0–1. If the value of PLI is 0, it means that there is no connectivity; if the value of PLI is 1, it means that there exists a complete phase lock value.

C. Pearson’s correlation: it is used to evaluate the correlation between two signals.

#### 2.3.4. The Construction of the Brain’s Static Complex Network

**Figure 1:**
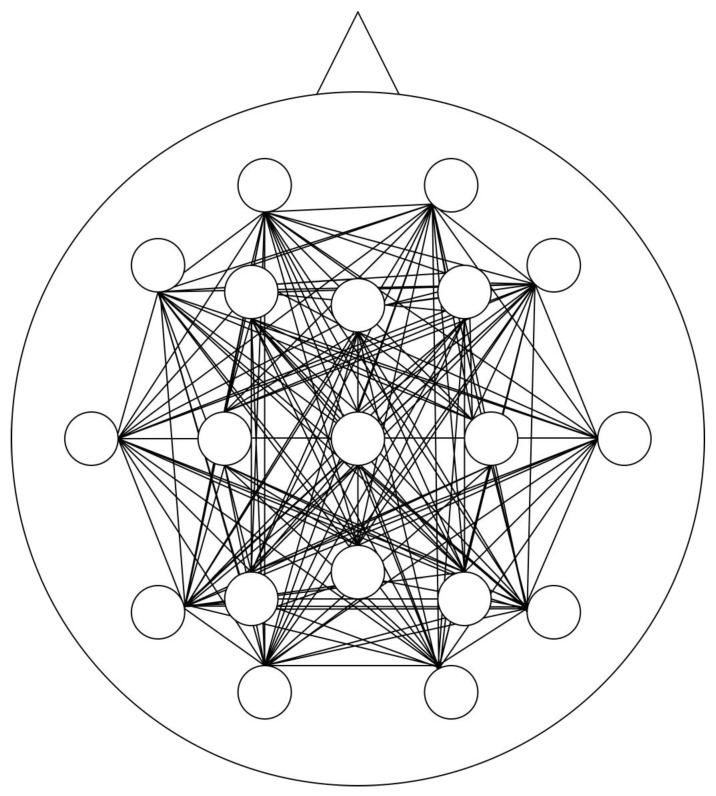
Schematic diagram of the brain’s complex network (full connection).

In the process of constructing the brain’s complex network with frequency-filtered EEG signals, each lead was regarded as a node. We used the following method to establish a complex network of the brain: if connectivity existed between leads, it was regarded as the existence of edges between nodes, and the connectivity strength between leads was regarded as the weight of the edges. EEGLAB Toolbox and FCLAB Toolbox functions were used for analyzing the static complex networks of the brain [22], and some self-edited MATLAB scripts were needed to build. The schematic diagram of the brain’s complex network (full connection) is shown in Figure 1.

#### 2.3.5. The Construction of the Brain’s Dynamic Complex Network

We used the sliding window method [23], selected a window whose width was 3 s, and used the window to shift repeatedly with the overlap 2/3 method (the step length is 1 s), on the frequency-filtered EEG signals to cut out multiple chronological time series (each time series was regarded as a snapshot of the brain activity). Then, we calculated the connectivity and connectivity strength between the EEG leads to construct a complex brain network under each snapshot point. We arranged all the brain’s complex networks in chronological order and realized the construction of the brain’s dynamic complex networks.

#### 2.3.6. Selection of the Threshold in the Complex Network

After using the above methods to construct a brain network, we must determine a threshold to remove weak connections [24]. In complex network research related to the brain, there was no universally accepted threshold selection principle, and most researchers determined the thresholds based on their own experience [16]. Some scholars also believed that the process of selecting the threshold should not destroy the graph’s connectivity. There should not be a situation where a node is not connected to any other node [25].

This research superimposed the brain’s average complex networks constructed by PEs or PCs to form two averaged complex networks. In the static complex network model, we averaged the complex network formed by multiple participants; in the dynamic complex network model, we averaged the complex network formed by multiple participants and multiple snapshot points. Then, we arranged each edge’s weight in the network from low to high. Then, we used the ratio of 0~100% to remove edges with the lowest weights and determine whether the network was connected under this ratio simultaneously. We kept the threshold when the proportion of removed edges was the largest. At the same time, the network was still connected to achieve the purpose of removing weak connections while ensuring the graph was connected. The averaged complex network mentioned here was only used for the selection of the threshold, rather than establishing the models as below.

### 2.4. Feature Extraction under the Brain’s Static and Dynamic Complex Network

#### 2.4.1. Feature Extraction under the Brain’s Static Complex Network

In graph theory, we usually use the following topological features to describe the characteristics of a graph. In a weighted graph, the vertex strength represents the sum of the weights of all edges connected to a node. The distance between two nodes in a graph is defined as the length of the shortest path between nodes. The diameter is the longest distance between all node-pairs in a graph. The average path length is defined as the average distances between all node-pairs in a graph.

The clustering coefficient (also called transitivity) is defined as the ratio of 3 times the number of the triangle in the graph to the number connected triples (3 nodes connected by 2 edges) to compare the group’s tightness.

Many complex networks in the real world are highly clustered, while the distance between most nodes is very short. The network with this characteristic is called a “small-world” network model [26]. The small-world index is defined as a random graph randomly generated for a known network and the number of nodes and edges that are the same as the known network, and we compare the clustering coefficient (*C/C_rand_*) and the average path length (*L/L_rand_*). If the clustering coefficient of the network is greater than the random graph and the average path length remains approximately the same, it is considered that the known network exhibits a small-world characteristic. That is, $S=\frac{\frac{C}{C_{rand}}}{\frac{L}{L_{rand}}}$, if *S* > 1, we consider that the network has the “small-world” characteristic.

Except for the calculation of small-world index, in which the self-write function was used, other topological features were calculated using functions in the “igraph” package in R.

#### 2.4.2. Feature Extraction under the Brain’s Dynamic Complex Network

Under each snapshot point, according to the mentioned brain’s complex network feature extraction methods, we analyzed the topological features of each constructed brain complex network, and we arranged the multiple features, formed by the snapshot points, in the time sequence to form multiple topological feature time series. For each formed topological feature time series, we calculated the mean, standard deviation, median, and interquartile range, respectively, as the features under the brain’s dynamic complex network.

### 2.5. Feature Selection, Machine Learning Classifier and Evaluating

#### 2.5.1. Feature Selection and Machine Learning Classifier

For the multiple features extracted from the static or dynamic complex network, we used the univariate analysis (*t*-test or Wilcoxon’s rank-sum test) method to compare the epilepsy patient group and the healthy control group and eliminated the insignificant features in the univariate analysis (*p* > 0.05). There were many features (≤20) in the dynamic complex network, and the principal component analysis (PCA) method was used to reduce the dimensionality, and the number of principal components was selected according to the results of the scree map. We incorporated features into classifiers of various machine learning models for discrimination. These classification models included logistic regression, decision tree, support vector machine, random forest, naïve bayes network, and bp neural network. We used default parameters in the above model.

#### 2.5.2. Evaluating the Classification Performances

We used the leave-one-out cross-validation (LOOCV) framework to calculate the total accuracy and the area under the ROC curve (*AUC*) of a model. Finally, we compared the result between the models without PCA and after PCA; the best result of each evaluation index was retained as the final evaluation result of a model.

**Figure 2:**
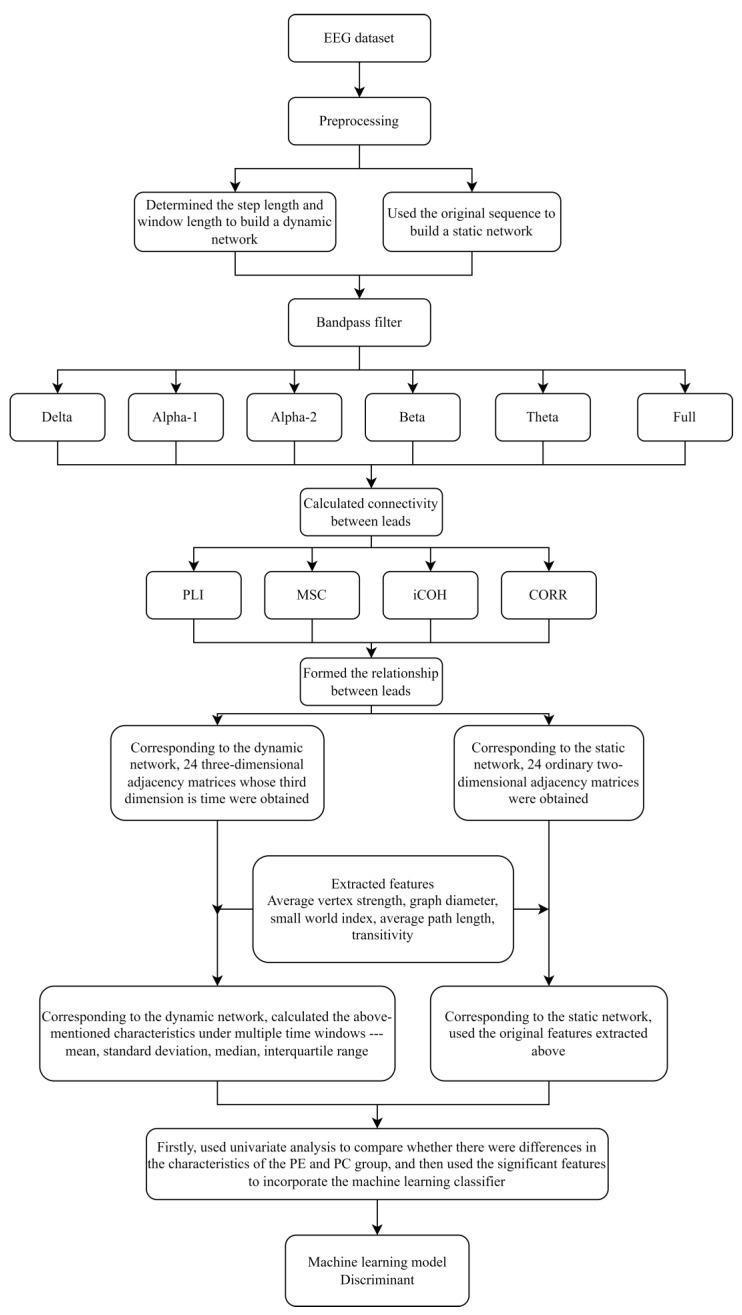
The roadmap of this research.

This research constructed a static brain complex network model, and at the same time, used the sliding window method to form a dynamic brain complex network model. We extracted the static features (the multiple topological features in the brain complex network) from the static brain complex network model and extracted the dynamic features from the dynamic brain complex network. Then, we incorporated features into the above machine learning models, and the total accuracy and *AUC* of the above two types of models under LOOCV were compared and analyzed. We aimed to evaluate the performance of the brain’s static and dynamic complex network models. The roadmap of this research is shown in Figure 2.

## 3. Results

### 3.1. Threshold Selection

**Figure 3:**
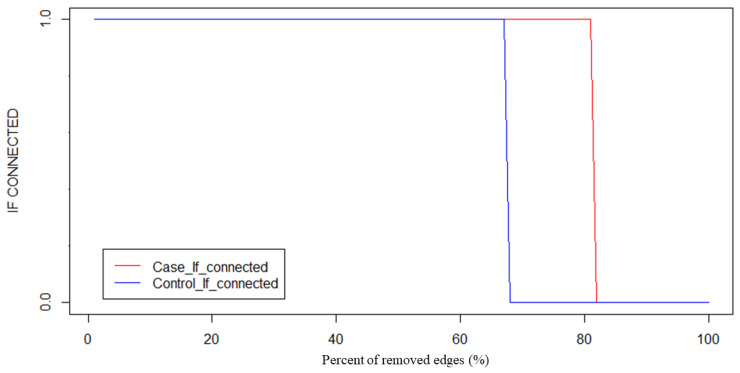
Threshold selection process (Example: under full-frequency band and PLI method).

In the two average networks formed by the superposition of the brain networks constructed by all PEs or PCs, the weight of each edge in the network was arranged from low to high and we used the ratio of 0~100% to remove edges with the lowest weights and determined whether the network was connected under this ratio at the same time. Take the PLI method in the full-frequency band as an example. As we can see from Figure 3, the network was disconnected when 65% of the edges were removed in the PC group, and the network was disconnected when 82% of the edges were removed in the PE group. Therefore, in both the PE group and the PC group, 64% of the edges of each network were removed while ensuring the connectivity of the graph.

### 3.2. Univariate Analysis Results

Here, we take the analysis under the full-frequency band as an example. As we can see, comparing with Table 2 and Table 3 or Table 4 and Table 5, the initially insignificant topological characteristics between the PE and the PC group in the brain static complex network could be transformed to significance, using the method of feature extraction under the brain’s dynamic complex network. See the Supplementary Parts S1–S4 for all results of six frequency bands with four connectivity methods.

**Figure 4:**
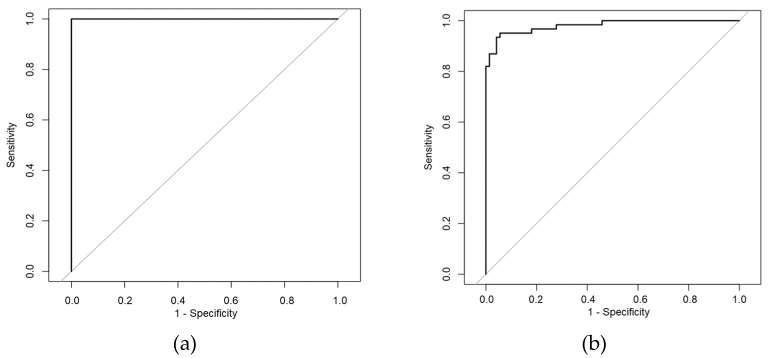
ROC curves with the highest accuracy rate under the two datasets. (**a**) iCOH method under the full-frequency band; dynamic network; original sequence EEG signals dataset; decision tree method; *AUC* = 1.000. (**b**) iCOH method under the full-frequency band; dynamic network; split segment EEG signals dataset; support vector machine method; *AUC* = 0.981.

Then, in the process of established machine learning classifiers mentioned above, under the connectivity calculation methods of MSC and iCOH between leads, the accuracies of using dynamic complex network features for discrimination were higher than that of static complex network features. Particularly in the iCOH method under the full-frequency band (in the original sequence EEG signals dataset), the discrimination accuracies of most machine learning classifiers were higher than 95%. Even in the expanded dataset (in the split segment EEG signals dataset), the discrimination accuracies of most machine learning models were higher than 90%. Furthermore, in the iCOH method under the full-frequency band, most models’ *AUC* were higher than 0.95 (in the original sequence EEG signals dataset or the split segment EEG signals dataset). Under the two data sets, the ROC curves at the highest *AUC* value are shown in Figure 4.

**Figure 5:**
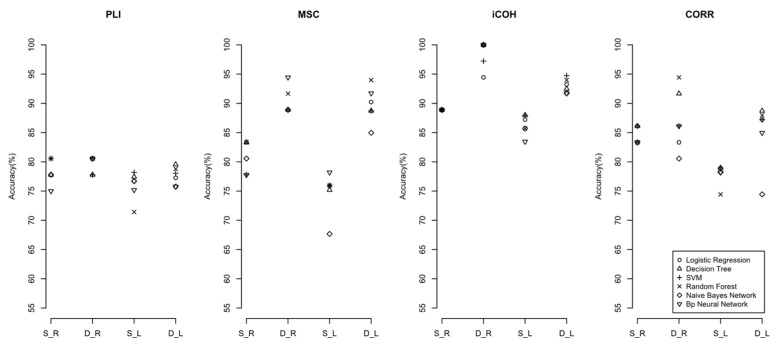
The accuracy of machine learning classifier with significant features under the full-frequency band (S: static network; D: dynamic network; L: split segment EEG signals dataset; R: original sequence EEG signals dataset; PLI: phase delay index; MSC: amplitude squared coherence; iCOH: coherence function Imaginary part; CORR: Pearson correlation coefficient).

**Figure 6:**
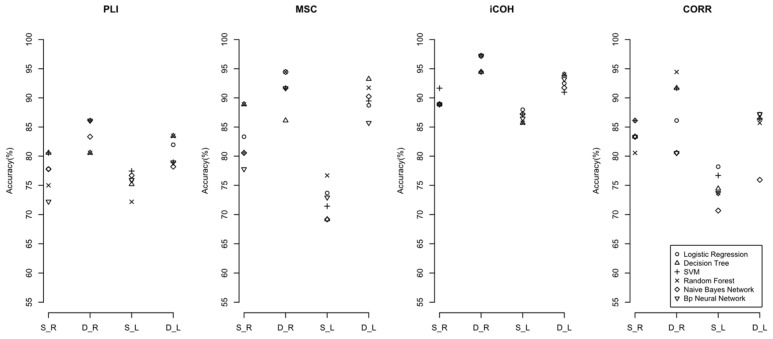
The accuracy of machine learning classifier with significant features under the beta-frequency band. (S: static network; D: dynamic network; L: split segment EEG signals dataset; R: original sequence EEG signals dataset; PLI: phase delay index; MSC: amplitude squared coherence; iCOH: coherence function Imaginary part; CORR: Pearson correlation coefficient).

In addition, the results in different frequency bands found that the discrimination accuracies of each machine learning model in the higher-frequency band (beta-frequency band) and the full-frequency band were higher than that of the lower-frequency bands. It means that the higher-frequency band of the EEG in PE patients may contain more useful information. Figure 5 and Figure 6 compare the accuracies of machine learning classifiers in the full-frequency and beta-frequency bands. See the Supplementary Part S5 for the comparisons of the accuracies in other frequency bands.

## 4. Discussion

### 4.1. Constructing the Brain’s Dynamic Complex Network and Extracting Its Characteristics Has a Better Performance than the Brain’s Static Complex Network

In this research, we used the sliding window method to construct the brain’s complex network, topological characteristics were calculated under multiple windows to form a time series, and the features of the time series were calculated as the characteristics of the dynamic network. As we incorporated the above dynamic characteristics into a variety of machine learning classifiers, it could be seen from the results that in most methods which involve the connectivity between leads, the accuracies of using dynamic characteristics for discrimination were higher than those of using static characteristics. Additionally, from the results of direct comparison of the features between the PC and PE groups, the initially insignificant topological characteristics between the PE and the PC group in the brain static complex network could be transformed to significant, using the method for feature extraction under the brain’s static complex network. In the iCOH method, the discrimination accuracies of most machine learning models were higher than 95%. This method has reached or even surpassed the level of the latest reports on the use of EEG signals in the framework of machine learning models to discriminate epilepsy patients and healthy controls in recent years [13,27,28], and it is a simple and intuitive feature extraction method compared with VG or recurrence network (RN) [29,30]. The time-varying characteristics existing in the EEG could be better explored [16,31,32]; compared with the results under the static complex network [27], the discrimination accuracies of the machine learning classifiers were also improved.

### 4.2. The Impact of EEG Frequency Band Splitting on the Discriminant Effect of Machine Learning Models

From the discriminant results under the six frequency bands, the discriminant accuracies of the higher-frequency band and the full-frequency band were the highest, indicating that the functional connection features contained in the EEG during epileptic seizures in patients with epilepsy were primarily concentrated in higher-frequency bands. This result is consistent with some previous studies [14,33]. Nevertheless, other epilepsy studies found that the alpha-band may play an important role [34,35]. In the research of using EEG signals to construct complex brain networks and to research functional connectivity, according to our results, the higher-frequency band’s characteristics may be considered to distinguish epilepsy patients and HCs.

### 4.3. Changes in the Topological Characteristics of PE in the Brain Network

It can be seen from the univariate analysis of characteristics that compared with the PC, the PE group had a larger small-world index, clustering coefficient, vertex strength, and transitivity in multiple dimensions (mean, standard deviation, median, interquartile range), indicating that the brain’s network of PE patients had a centralized tendency during the ictal phase. It can be considered that the connection between some leads becomes stronger, forming a closely connected community structure, the electrical activity is conducted rapidly, and in the brain, there may exist a closely connected region. This may be related to the conduction of abnormal discharge through neurons and the synchronous discharge of peripheral and distant neurons during epilepsy; this result is consistent with previous studies on the topological characteristics of the brain’s function connections with epilepsy [16,36,37].

### 4.4. Shortage of This Research

In this research, the sample size used was relatively small. As we enriched the sample size and avoided over-fitting in the machine learning model, this research split the signals with longer EEG records in the two groups (PE and PC) to make the sample size larger. However, after this step, there were multiple fragments from the same subject to be correlated. We would consider a larger sample size to establish a more robust machine learning classifier in further research. In addition, only simple features (mean, variance, median, interquartile range) were considered in the process of character extraction of the time series formed by topological features. In further research, we would consider other time series features to classify and discriminate by various machine learning methods. Under the sliding window method, our research’s determination of window width was still subjective. A shorter window width may be more affected by noise, while a more extended window width may conceal the rapidly changing information in the EEG [38]. However, scholars have only advised the window widths for dynamic networks constructed with the sliding windows method under fMRI [39]. In the following research, we will try a variety of window widths and step length combinations to determine the most suitable parameters under EEG for discovering dynamic features.

## 5. Conclusions

This research proposed a framework applied to the EEG of pediatric epilepsy during the ictal phase. We used the sliding window method and multiple functional connection indicators to construct the dynamic complex network of the brain and extract dynamic features into various machine learning classifiers for discrimination. The results showed that, compared with the static features extracted under the traditionally constructed brain’s static complex network, this research can successfully summarize more time-varying features in the EEG and improve the accuracy of the discrimination of the machine learning classifier, solely using static features. This framework is worthy of its expected higher efficiency in its application on other diseases.

## Figures and Tables

**Table 1:** The demographic characteristics of participants.

Factor	PE	PC
Age (years)	7.75 ± 4.92	7.05 ± 3.53
Gender *n* (%)		
Female	6 (37.5)	7 (35.0)
Male	10 (62.5)	13 (65.0)

**Table 2:** Univariate analysis of static network characteristics.

Item	*P*_50__PC	*IQR*_PC	*P*_50__PE	*IQR*_PE	*W*	*p*
Small-world index	1.21	0.21	1.33	0.28	106.0	0.089
Average vertex strength	0.59	0.16	1.03	0.62	53.0	<0.001 *
Average path length	1.63	0.04	1.64	0.05	136.0	0.453
Transitivity	0.44	0.04	0.47	0.07	111.0	0.124
Diameter	0.22	0.07	0.36	0.27	67.0	0.002 *

(Original sequence EEG signals dataset; PLI as the connectivity method in full-frequency band, rank-sum test was used to compare the difference as normality was not satisfied, * *p* < 0.05).

**Table 3:** Univariate analysis of dynamic network characteristics.

Item	*P*_50__PC	*IQR*_PC	*P*_50__PE	*IQR*_PE	*W*	*p*
*Mean*-Small-world index	1.13	0.03	1.16	0.05	84.0	0.015 *
*Standard Deviation*-Small-world index	0.18	0.02	0.18	0.01	117.0	0.178
*P*_50_-Small-world index	1.12	0.05	1.14	0.04	83.0	0.014 *
*IQR*-Small-world index	0.24	0.05	0.25	0.05	132.0	0.386
*Mean*-Average vertex strength	2.60	0.23	2.91	0.33	65.0	0.002 *
*Standard Deviation*-Average vertex strength	0.38	0.12	0.49	0.22	53.0	<0.001 *
*P*_50_-Average vertex strength	2.59	0.26	2.82	0.17	63.0	0.002 *
*IQR*-Average vertex strength	0.51	0.18	0.66	0.30	43.0	<0.001 *
*Mean*-Average path length	1.64	0.01	1.64	0.01	75.0	0.006 *
*Standard Deviation*-Average path length	0.03	0.01	0.04	0.01	84.0	0.015 *
*P_50_*-Average path length	1.63	0.01	1.64	0.01	44.0	<0.001 *
*IQR*-Average path length	0.04	0.01	0.05	0.02	86.5	0.019 *
*Mean*-Transitivity	0.44	0.01	0.45	0.02	60.0	0.001 *
*Standard Deviation*-Transitivity	0.04	0.01	0.05	0.00	55.0	0.001 *
*P*_50_-Transitivity	0.44	0.01	0.45	0.02	62.5	0.002 *
*IQR*-Transitivity	0.06	0.01	0.07	0.01	71.0	0.004 *
*Mean*-Diameter	0.96	0.09	1.09	0.14	57.0	0.001 *
*Standard Deviation*-Diameter	0.19	0.05	0.26	0.10	60.0	0.001 *
*P*_50_-Diameter	0.94	0.08	1.04	0.12	55.5	0.001 *
*IQR*-Diameter	0.23	0.08	0.32	0.11	44.0	<0.001 *

(Original sequence EEG signals dataset; PLI as the connectivity method in full-frequency band, the rank-sum test was used to compare the difference as normality was not satisfied, * *p* < 0.05).

**Table 4:** Univariate analysis of static network characteristics.

Item	*P*_50__PC	*IQR*_PC	*P*_50__PE	*IQR*_PE	*W*	*p*
Small-world index	1.22	0.23	1.29	0.29	1977.0	0.324
Average vertex strength	0.64	0.24	0.97	0.72	912.0	<0.001 *
Average path length	1.64	0.04	1.64	0.05	2487.5	0.188
Transitivity	0.44	0.06	0.46	0.08	1864.0	0.134
Diameter	0.24	0.10	0.36	0.26	1031.5	<0.001 *

(Split segment EEG signals dataset; PLI as the connectivity method in full-frequency band, the rank-sum test was used to compare the difference as normality was not satisfied, * *p* < 0.05).

**Table 5:** Univariate analysis of dynamic network characteristics.

Item	*P*_50__PC	*IQR*_PC	*P_50_*_PE	*IQR*_PE	*W*	*p*
*Mean*-Small-world index	1.14	0.03	1.15	0.05	1658.0	0.015 *
*Standard Deviation*-Small-world index	0.17	0.02	0.18	0.03	1421.0	<0.001 *
*P*_50_-Small-world index	1.13	0.05	1.14	0.05	1642.0	0.012 *
*IQR*-Small-world index	0.23	0.04	0.24	0.05	1638.0	0.012 *
*Mean*-Average vertex strength	2.60	0.22	2.84	0.39	1008.0	<0.001 *
*Standard Deviation*-Average vertex strength	0.39	0.11	0.49	0.18	897.0	<0.001 *
*P_50_*-Average vertex strength	2.55	0.23	2.80	0.29	1030.5	<0.001 *
*IQR*-Average vertex strength	0.52	0.11	0.65	0.30	843.5	<0.001 *
*Mean*-Average path length	1.64	0.01	1.64	0.01	1751.0	0.045 *
*Standard Deviation*-Average path length	0.04	0.01	0.04	0.01	1143.0	<0.001 *
*P_50_*-Average path length	1.63	0.01	1.64	0.01	1503.5	0.001 *
*IQR*-Average path length	0.04	0.01	0.05	0.01	1540.0	0.003 *
*Mean*-Transitivity	0.44	0.01	0.45	0.01	1072.0	<0.001 *
*Standard Deviation*-Transitivity	0.05	0.01	0.05	0.01	1354.0	<0.001 *
*P*_50_-Transitivity	0.44	0.01	0.45	0.01	1100.0	<0.001 *
*IQR*-Transitivity	0.06	0.01	0.06	0.01	1576.0	0.005 *
*Mean*-Diameter	0.96	0.09	1.06	0.17	947.0	<0.001 *
*Standard Deviation*-Diameter	0.20	0.05	0.24	0.09	934.0	<0.001 *
*P_50_*-Diameter	0.93	0.09	1.03	0.14	903.5	<0.001 *
*IQR*-Diameter	0.25	0.07	0.31	0.11	898.0	<0.001 *

(Split segment EEG signals dataset; PLI as the connectivity method in full-frequency band, the rank-sum test was used to compare the difference as normality was not satisfied, * *p* < 0.05).

## Data Availability

The data presented in this study are available on request from the corresponding author. The data are not publicly available due to issues of participant confidentiality.

## Supplementary Materials

The following supporting information can be downloaded at: https://www.mdpi.com/article/10.3390/s22072553/s1, Supplementary Part S1: Univariate analysis of static network characteristics (Original sequence EEG signals dataset). 24 tables (a combination of 4 connectivity methods with 6 frequency bands); Supplementary Part S2: Univariate analysis of static network characteristics (Split segment EEG signals dataset). 24 tables (a combination of 4 connectivity methods with 6 frequency bands); Supplementary Part S3: Univariate analysis of dynamic network characteristics (Original sequence EEG signals dataset). 24 tables (a combination of 4 connectivity methods with 6 frequency bands); Supplementary Part S4: Univariate analysis of dynamic network characteristics (Split segment EEG signals dataset). 24 tables (a combination of 4 connectivity methods with 6 frequency bands); Supplementary Part S5: The accuracy of machine learning classifiers with significant features. 6 figures (under 6 frequency bands).
