# Supplementary material for: Feature Extraction of the Brain’s Dynamic Complex Network Based on EEG and a Framework for Discrimination of Pediatric Epilepsy
Source: Sensors (Basel). 2022 Mar 26;22(7):2553. doi: 10.3390/s22072553 (PMC9003013; doi:10.3390/s22072553)
Supplement: Supplementary file 1 [file sensors-22-02553-s001.zip › sensors-1656130-supplementary.pdf]

Supplementary Part S1

Univariate analysis of static network characteristics-Original sequence EEG signals dataset

Table S1-1: Univariate analysis of static network characteristic

Item	P_{50}_PC	*IQR*_PC	P_{50}_PE	*IQR*_PE	*W*	*P*
Little world index	1.21	0.21	1.33	0.28	106.0	0.089
Average vertex strength	0.59	0.16	1.03	0.62	53.0	<0.001*
Average path length	1.63	0.04	1.64	0.05	136.0	0.453
Transitivity	0.44	0.04	0.47	0.07	111.0	0.124
Diameter	0.22	0.07	0.36	0.27	67.0	0.002*

(PLI as the connectivity method in Delta frequency band, the rank-sum test was used to compare the difference as normality was not satisfied, * $P<0.05$)

Table S1-2: Univariate analysis of static network characteristic

Item	P_{50}_PC	*IQR*_PC	P_{50}_PE	*IQR*_PE	*W*	*P*
Little world index	0.94	0.20	1.02	0.14	98.0	0.049*
Average vertex strength	3.96	0.39	4.39	0.46	65.0	0.002*
Average path length	1.87	0.14	1.86	0.09	178.5	0.566
Transitivity	0.54	0.07	0.55	0.10	142.0	0.582
Diameter	1.16	0.50	1.17	0.29	133.0	0.404

(MSC as the connectivity method in Delta frequency band, the rank-sum test was used to compare the difference as normality was not satisfied, * $P<0.05$)

Table S1-3: Univariate analysis of static network characteristic

Item	P_{50}_PC	*IQR*_PC	P_{50}_PE	*IQR*_PE	*W*	*P*
Little world index	1.02	0.21	1.14	0.24	125.0	0.276
Average vertex strength	0.48	0.26	0.75	0.43	68.0	0.003*
Average path length	1.58	0.03	1.58	0.05	170.5	0.750
Transitivity	0.46	0.06	0.48	0.07	122.0	0.236
Diameter	0.15	0.07	0.23	0.12	67.0	0.002*

(iCOH as the connectivity method in Delta frequency band, the rank-sum test was used to compare the difference as normality was not satisfied, * $P<0.05$)

Table S1-4: Univariate analysis of static network characteristic

Item	P_{50}_PC	*IQR*_PC	P_{50}_PE	*IQR*_PE	*W*	*P*
Little world index	0.96	0.16	1.03	0.24	101.0	0.062
Average vertex strength	5.37	0.42	5.63	0.39	71.0	0.004*
Average path length	1.87	0.14	1.83	0.12	194.5	0.279
Transitivity	0.56	0.10	0.55	0.12	164.0	0.912
Diameter	2.01	0.72	1.99	0.42	152.0	0.814

(CORR as the connectivity method in Delta frequency band, the rank-sum test was used to compare the difference as normality was not satisfied, * $P<0.05$)

Table S1-5: Univariate analysis of static network characteristic

Item	P_{50}_PC	*IQR*_PC	P_{50}_PE	*IQR*_PE	*W*	*P*
Little world index	1.25	0.28	1.25	0.30	167.0	0.838
Average vertex strength	0.53	0.19	1.00	0.56	43.0	<0.001*
Average path length	1.63	0.03	1.62	0.07	199.5	0.213
Transitivity	0.46	0.06	0.47	0.09	155.0	0.888
Diameter	0.19	0.07	0.36	0.22	55.0	0.001*

(PLI as the connectivity method in Theta frequency band, the rank-sum test was used to compare the difference as normality was not satisfied, * P<0.05)

Table S1-6: Univariate analysis of static network characteristic

Item	P_{50}_PC	*IQR*_PC	P_{50}_PE	*IQR*_PE	*W*	*P*
Little world index	0.99	0.14	0.97	0.15	142.0	0.582
Average vertex strength	3.89	0.54	4.50	0.63	44.0	<0.001*
Average path length	1.86	0.14	1.84	0.08	167.5	0.824
Transitivity	0.55	0.11	0.55	0.08	143.5	0.610
Diameter	1.00	0.41	1.37	0.38	75.0	0.006*

(MSC as the connectivity method in Theta frequency band, the rank-sum test was used to compare the difference as normality was not satisfied, * P<0.05)

Table S1-7: Univariate analysis of static network characteristic

Item	P_{50}_PC	*IQR*_PC	P_{50}_PE	*IQR*_PE	*W*	*P*
Little world index	1.11	0.14	1.10	0.27	177.0	0.604
Average vertex strength	0.51	0.32	1.09	0.64	36.0	<0.001*
Average path length	1.57	0.04	1.58	0.04	151.0	0.786
Transitivity	0.48	0.06	0.46	0.07	201.0	0.200
Diameter	0.16	0.10	0.35	0.18	31.0	<0.001*

(iCOH as the connectivity method in Theta frequency band, the rank-sum test was used to compare the difference as normality was not satisfied, * P<0.05)

Table S1-8: Univariate analysis of static network characteristic

Item	P_{50}_PC	*IQR*_PC	P_{50}_PE	*IQR*_PE	*W*	*P*
Little world index	0.95	0.16	0.98	0.14	129.0	0.336
Average vertex strength	5.33	0.25	5.71	0.32	520.0	<0.001*
Average path length	1.86	0.11	1.85	0.10	167.0	0.836
Transitivity	0.55	0.07	0.53	0.12	162.0	0.962
Diameter	1.99	0.45	2.12	0.41	118.0	0.189

(CORR as the connectivity method in Theta frequency band, the rank-sum test was used to compare the difference as normality was not satisfied, * P<0.05)

Table S1-9: Univariate analysis of static network characteristic

Item	P_{50}_PC	*IQR*_PC	P_{50}_PE	*IQR*_PE	*W*	*P*
Little world index	1.33	0.29	1.23	0.38	192.0	0.320
Average vertex strength	0.51	0.19	1.00	0.56	40.0	<0.001*
Average path length	1.63	0.02	1.63	0.04	163.5	0.924
Transitivity	0.47	0.05	0.45	0.11	185.0	0.440
Diameter	0.18	0.09	0.36	0.20	51.0	<0.001*

(PLI as the connectivity method in Alpha-1 frequency band, the rank-sum test was used to compare the difference as normality was not satisfied, * P<0.05)

Table S1-10: Univariate analysis of static network characteristic

Item	P_{50}_PC	*IQR*_PC	P_{50}_PE	*IQR*_PE	*W*	*P*
Little world index	1.00	0.22	1.04	0.18	130.0	0.352
Average vertex strength	3.93	0.86	4.45	0.71	89.0	0.023*
Average path length	1.82	0.14	1.80	0.09	192.5	0.308
Transitivity	0.55	0.14	0.56	0.14	145.0	0.648
Diameter	1.15	0.57	1.19	0.38	136.0	0.459

(MSC as the connectivity method in Alpha-1 frequency band, the rank-sum test was used to compare the difference as normality was not satisfied, * P<0.05)

Table S1-11: Univariate analysis of static network characteristic

Item	P_{50}_PC	*IQR*_PC	P_{50}_PE	*IQR*_PE	*W*	*P*
Little world index	1.12	0.21	1.13	0.34	143.0	0.604
Average vertex strength	0.77	0.23	1.21	0.80	44.0	<0.001*
Average path length	1.57	0.02	1.57	0.03	161.5	0.974
Transitivity	0.48	0.08	0.47	0.06	155.0	0.888
Diameter	0.23	0.07	0.35	0.29	55.0	0.001*

(iCOH as the connectivity method in Alpha-1 frequency band, the rank-sum test was used to compare the difference as normality was not satisfied, * P<0.05)

Table S1-12: Univariate analysis of static network characteristic

Item	P_{50}_PC	*IQR*_PC	P_{50}_PE	*IQR*_PE	*W*	*P*
Little world index	0.93	0.12	0.96	0.16	127.0	0.305
Average vertex strength	5.32	0.19	5.72	0.30	44.0	<0.001*
Average path length	1.93	0.12	1.85	0.13	205.5	0.152
Transitivity	0.56	0.08	0.54	0.11	161.0	0.987
Diameter	2.10	0.65	2.12	0.41	144.0	0.626

(CORR as the connectivity method in Alpha-1 frequency band, the rank-sum test was used to compare the difference as normality was not satisfied, * P<0.05)

Table S1-13: Univariate analysis of static network characteristic

Item	P_{50}_PC	*IQR*_PC	P_{50}_PE	*IQR*_PE	*W*	*P*
Little world index	1.33	0.28	1.34	0.30	177.0	0.604
Average vertex strength	0.52	0.19	0.98	0.56	44.0	<0.001*
Average path length	1.63	0.04	1.63	0.04	184.5	0.444
Transitivity	0.47	0.07	0.44	0.08	199.0	0.223
Diameter	0.18	0.07	0.35	0.19	53.0	<0.001*

(PLI as the connectivity method in Alpha-2 frequency band, the rank-sum test was used to compare the difference as normality was not satisfied, * $P<0.05$)

Table S1-14: Univariate analysis of static network characteristic

Item	P_{50}_PC	*IQR*_PC	P_{50}_PE	*IQR*_PE	*W*	*P*
Little world index	1.05	0.14	1.04	0.14	134.0	0.422
Average vertex strength	3.95	0.88	4.53	0.58	91.0	0.028*
Average path length	1.83	0.10	1.82	0.12	171.0	0.738
Transitivity	0.56	0.16	0.59	0.12	136.0	0.459
Diameter	1.07	0.50	1.35	0.47	104.0	0.077

(MSC as the connectivity method in Alpha-2 frequency band, the rank-sum test was used to compare the difference as normality was not satisfied, * $P<0.05$)

Table S1-15: Univariate analysis of static network characteristic

Item	P_{50}_PC	*IQR*_PC	P_{50}_PE	*IQR*_PE	*W*	*P*
Little world index	1.14	0.22	1.13	0.26	173.0	0.694
Average vertex strength	0.83	0.67	1.42	0.73	73.0	0.005*
Average path length	1.57	0.02	1.58	0.03	154.0	0.860
Transitivity	0.47	0.05	0.50	0.10	125.0	0.276
Diameter	0.28	0.19	0.44	0.20	77.0	0.007*

(iCOH as the connectivity method in Alpha-2 frequency band, the rank-sum test was used to compare the difference as normality was not satisfied, * $P<0.05$)

Table S1-16: Univariate analysis of static network characteristic

Item	P_{50}_PC	*IQR*_PC	P_{50}_PE	*IQR*_PE	*W*	*P*
Little world index	0.94	0.11	0.97	0.15	123.0	0.249
Average vertex strength	5.30	0.20	5.71	0.32	45.0	<0.001*
Average path length	1.89	0.13	1.85	0.12	191.5	0.324
Transitivity	0.55	0.08	0.53	0.11	161.0	0.987
Diameter	2.00	0.57	2.12	0.40	131.0	0.369

(CORR as the connectivity method in Alpha-2 frequency band, the rank-sum test was used to compare the difference as normality was not satisfied, * $P<0.05$)

Table S1-17: Univariate analysis of static network characteristic

Item	P_{50}_PC	*IQR*_PC	P_{50}_PE	*IQR*_PE	*W*	*P*
Little world index	1.38	0.23	1.35	0.35	178.0	0.582
Average vertex strength	0.49	0.18	0.94	0.54	37.0	<0.001*
Average path length	1.64	0.03	1.63	0.04	186.5	0.407
Transitivity	0.47	0.05	0.46	0.09	169.0	0.789
Diameter	0.18	0.08	0.35	0.21	42.0	<0.001*

(PLI as the connectivity method in Beta frequency band, the rank-sum test was used to compare the difference as normality was not satisfied, * $P<0.05$)

Table S1-18: Univariate analysis of static network characteristic

Item	P_{50}_PC	*IQR*_PC	P_{50}_PE	*IQR*_PE	*W*	*P*
Little world index	0.95	0.12	0.99	0.21	123.0	0.249
Average vertex strength	3.18	0.31	3.62	0.67	40.0	<0.001*
Average path length	1.82	0.07	1.83	0.10	137.0	0.474
Transitivity	0.50	0.08	0.54	0.10	112.5	0.135
Diameter	0.61	0.20	1.01	0.44	45.0	<0.001*

(MSC as the connectivity method in Beta frequency band, the rank-sum test was used to compare the difference as normality was not satisfied, * $P<0.05$)

Table S1-19: Univariate analysis of static network characteristic

Item	P_{50}_PC	*IQR*_PC	P_{50}_PE	*IQR*_PE	*W*	*P*
Little world index	1.07	0.16	1.16	0.22	105.0	0.083
Average vertex strength	0.30	0.13	0.85	0.58	7.0	<0.001*
Average path length	1.58	0.04	1.57	0.04	190.0	0.346
Transitivity	0.47	0.04	0.48	0.06	118.0	0.189
Diameter	0.09	0.04	0.25	0.19	13.0	<0.001*

(iCOH as the connectivity method in Beta frequency band, the rank-sum test was used to compare the difference as normality was not satisfied, * $P<0.05$)

Table S1-20: Univariate analysis of static network characteristic

Item	P_{50}_PC	*IQR*_PC	P_{50}_PE	*IQR*_PE	*W*	*P*
Little world index	0.93	0.13	1.00	0.18	104.0	0.077
Average vertex strength	5.27	0.19	5.65	0.32	41.0	<0.001*
Average path length	1.88	0.14	1.84	0.09	206.5	0.143
Transitivity	0.55	0.08	0.53	0.10	157.0	0.937
Diameter	2.03	0.48	2.08	0.33	129.0	0.336

(CORR as the connectivity method in Beta frequency band, the rank-sum test was used to compare the difference as normality was not satisfied, * $P<0.05$)

Table S1-21: Univariate analysis of static network characteristic

Item	P_{50}_PC	*IQR*_PC	P_{50}_PE	*IQR*_PE	*W*	*P*
Little world index	1.36	0.22	1.30	0.36	170.0	0.765
Average vertex strength	0.49	0.17	0.94	0.54	38.0	<0.001*
Average path length	1.64	0.03	1.62	0.05	214.0	0.087
Transitivity	0.47	0.03	0.46	0.09	166.0	0.861
Diameter	0.18	0.07	0.35	0.21	40.0	<0.001*

(PLI as the connectivity method in full frequency band, the rank-sum test was used to compare the difference as normality was not satisfied, * P<0.05)

Table S1-22: Univariate analysis of static network characteristic

Item	P_{50}_PC	*IQR*_PC	P_{50}_PE	*IQR*_PE	*W*	*P*
Little world index	0.97	0.14	0.99	0.15	122.0	0.236
Average vertex strength	3.35	0.28	3.73	0.36	34.0	<0.001*
Average path length	1.83	0.10	1.80	0.11	177.5	0.588
Transitivity	0.53	0.08	0.51	0.09	157.0	0.937
Diameter	0.65	0.22	0.87	0.29	55.0	0.001*

(MSC as the connectivity method in full frequency band, the rank-sum test was used to compare the difference as normality was not satisfied, * P<0.05)

Table S1-23: Univariate analysis of static network characteristic

Item	P_{50}_PC	*IQR*_PC	P_{50}_PE	*IQR*_PE	*W*	*P*
Little world index	1.08	0.16	1.14	0.21	135.0	0.440
Average vertex strength	0.23	0.11	0.63	0.36	10.0	<0.001*
Average path length	1.57	0.03	1.58	0.03	133.0	0.396
Transitivity	0.47	0.04	0.48	0.07	136.0	0.459
Diameter	0.07	0.04	0.20	0.11	6.0	<0.001*

(iCOH as the connectivity method in full frequency band, the rank-sum test was used to compare the difference as normality was not satisfied, * P<0.05)

Table S1-24: Univariate analysis of static network characteristic

Item	P_{50}_PC	*IQR*_PC	P_{50}_PE	*IQR*_PE	*W*	*P*
Little world index	0.93	0.12	1.00	0.18	105.0	0.083
Average vertex strength	5.26	0.19	5.65	0.32	41.0	<0.001*
Average path length	1.88	0.13	1.84	0.09	207.5	0.134
Transitivity	0.55	0.08	0.53	0.10	160.0	1.000
Diameter	2.02	0.44	2.08	0.33	128.0	0.320

(CORR as the connectivity method in full frequency band, the rank-sum test was used to compare the difference as normality was not satisfied, * P<0.05)

Supplementary Part S2

Univariate analysis of static network characteristics-Split segment EEG signals dataset

Table S2-1: Univariate analysis of static network characteristic

Item	P_{50}_PC	*IQR*_PC	P_{50}_PE	*IQR*_PE	*W*	*P*
Little world index	1.22	0.23	1.29	0.29	1977.0	0.324
Average vertex strength	0.64	0.24	0.97	0.72	912.0	<0.001*
Average path length	1.64	0.04	1.64	0.05	2487.5	0.188
Transitivity	0.44	0.06	0.46	0.08	1864.0	0.134
Diameter	0.24	0.10	0.36	0.26	1031.5	<0.001*

(PLI as the connectivity method in Delta frequency band, the rank-sum test was used to compare the difference as normality was not satisfied, * $P<0.05$)

Table S2-2: Univariate analysis of static network characteristic

Item	P_{50}_PC	*IQR*_PC	P_{50}_PE	*IQR*_PE	*W*	*P*
Little world index	0.96	0.18	1.04	0.16	1582.0	0.006*
Average vertex strength	3.96	0.31	4.30	0.54	1046.0	<0.001*
Average path length	1.87	0.14	1.83	0.06	2678.0	0.030*
Transitivity	0.57	0.09	0.54	0.08	2720.0	0.018*
Diameter	1.11	0.35	1.20	0.36	1703.0	0.026*

(MSC as the connectivity method in Delta frequency band, the rank-sum test was used to compare the difference as normality was not satisfied, * $P<0.05$)

Table S2-3: Univariate analysis of static network characteristic

Item	P_{50}_PC	*IQR*_PC	P_{50}_PE	*IQR*_PE	*W*	*P*
Little world index	1.05	0.21	1.13	0.27	1826.0	0.095
Average vertex strength	0.51	0.26	0.81	0.73	956.0	<0.001*
Average path length	1.58	0.03	1.57	0.03	2501.0	0.168
Transitivity	0.46	0.05	0.47	0.09	1914.5	0.204
Diameter	0.16	0.09	0.25	0.18	1021.0	<0.001*

(iCOH as the connectivity method in Delta frequency band, the rank-sum test was used to compare the difference as normality was not satisfied, * $P<0.05$)

Table S2-4: Univariate analysis of static network characteristic

Item	P_{50}_PC	*IQR*_PC	P_{50}_PE	*IQR*_PE	*W*	*P*
Little world index	0.98	0.18	1.03	0.19	1705.0	0.027*
Average vertex strength	5.35	0.28	5.59	0.43	1284.0	<0.001*
Average path length	1.86	0.14	1.83	0.08	2599.5	0.069
Transitivity	0.56	0.10	0.54	0.07	2754.0	0.012*
Diameter	1.99	0.60	1.94	0.48	2150.0	0.837

(CORR as the connectivity method in Delta frequency band, the rank-sum test was used to compare the difference as normality was not satisfied, * $P<0.05$)

Table S2-5: Univariate analysis of static network characteristic

Item	P_{50}_PC	*IQR*_PC	P_{50}_PE	*IQR*_PE	*W*	*P*
Little world index	1.24	0.22	1.30	0.27	1975.0	0.319
Average vertex strength	0.57	0.25	0.93	0.52	784.0	<0.001*
Average path length	1.64	0.05	1.63	0.05	2607.0	0.063
Transitivity	0.45	0.07	0.46	0.06	2018.0	0.423
Diameter	0.20	0.11	0.35	0.21	856.5	<0.001*

(PLI as the connectivity method in Theta frequency band, the rank-sum test was used to compare the difference as normality was not satisfied, * P<0.05)

Table S2-6: Univariate analysis of static network characteristic

Item	P_{50}_PC	*IQR*_PC	P_{50}_PE	*IQR*_PE	*W*	*P*
Little world index	0.99	0.13	1.02	0.12	1836.0	0.105
Average vertex strength	3.91	0.47	4.45	0.62	1017.0	<0.001*
Average path length	1.82	0.11	1.86	0.09	1886.0	0.162
Transitivity	0.54	0.10	0.56	0.08	1961.5	0.291
Diameter	1.00	0.40	1.29	0.44	1195.0	<0.001*

(MSC as the connectivity method in Theta frequency band, the rank-sum test was used to compare the difference as normality was not satisfied, * P<0.05)

Table S2-7: Univariate analysis of static network characteristic

Item	P_{50}_PC	*IQR*_PC	P_{50}_PE	*IQR*_PE	*W*	*P*
Little world index	1.06	0.19	1.07	0.23	2102.0	0.673
Average vertex strength	0.61	0.37	1.13	0.57	721.0	<0.001*
Average path length	1.57	0.02	1.58	0.04	1849.0	0.116
Transitivity	0.45	0.06	0.47	0.07	2066.0	0.559
Diameter	0.18	0.11	0.35	0.18	747.0	<0.001*

(iCOH as the connectivity method in Theta frequency band, the rank-sum test was used to compare the difference as normality was not satisfied, * P<0.05)

Table S2-8: Univariate analysis of static network characteristic

Item	P_{50}_PC	*IQR*_PC	P_{50}_PE	*IQR*_PE	*W*	*P*
Little world index	0.97	0.17	1.00	0.15	1870.0	0.142
Average vertex strength	5.32	0.25	5.59	0.41	1105.0	<0.001*
Average path length	1.86	0.14	1.84	0.10	2412.5	0.329
Transitivity	0.56	0.08	0.53	0.08	2795.0	0.007*
Diameter	1.98	0.54	1.99	0.55	1990.0	0.353

(CORR as the connectivity method in Theta frequency band, the rank-sum test was used to compare the difference as normality was not satisfied, * P<0.05)

Table S2-9: Univariate analysis of static network characteristic

Item	P_{50}_PC	*IQR*_PC	P_{50}_PE	*IQR*_PE	*W*	*P*
Little world index	1.29	0.33	1.28	0.33	2193.0	0.991
Average vertex strength	0.59	0.23	0.93	0.50	840.0	<0.001*
Average path length	1.63	0.04	1.63	0.05	2291.5	0.668
Transitivity	0.46	0.07	0.45	0.07	2348.5	0.492
Diameter	0.21	0.11	0.34	0.20	879.0	<0.001*

(PLI as the connectivity method in Alpha-1 frequency band, the rank-sum test was used to compare the difference as normality was not satisfied, * $P<0.05$)

Table S2-10: Univariate analysis of static network characteristic

Item	P_{50}_PC	*IQR*_PC	P_{50}_PE	*IQR*_PE	*W*	*P*
Little world index	1.03	0.18	1.06	0.14	1865.0	0.136
Average vertex strength	3.98	0.84	4.44	0.81	1521.0	0.002*
Average path length	1.83	0.12	1.82	0.14	2362.5	0.453
Transitivity	0.55	0.12	0.58	0.14	1957.0	0.281
Diameter	1.10	0.62	1.18	0.46	1881.0	0.156

(MSC as the connectivity method in Alpha-1 frequency band, the rank-sum test was used to compare the difference as normality was not satisfied, * $P<0.05$)

Table S2-11: Univariate analysis of static network characteristic

Item	P_{50}_PC	*IQR*_PC	P_{50}_PE	*IQR*_PE	*W*	*P*
Little world index	1.07	0.22	1.08	0.34	2152.0	0.844
Average vertex strength	0.78	0.38	1.35	1.01	767.0	<0.001*
Average path length	1.57	0.03	1.57	0.05	2324.0	0.563
Transitivity	0.46	0.06	0.47	0.08	1940.0	0.249
Diameter	0.24	0.12	0.40	0.31	832.0	<0.001*

(iCOH as the connectivity method in Alpha-1 frequency band, the rank-sum test was used to compare the difference as normality was not satisfied, * $P<0.05$)

Table S2-12: Univariate analysis of static network characteristic

Item	P_{50}_PC	*IQR*_PC	P_{50}_PE	*IQR*_PE	*W*	*P*
Little world index	0.97	0.14	0.98	0.16	1907.0	0.193
Average vertex strength	5.33	0.21	5.58	0.40	1072.0	<0.001*
Average path length	1.86	0.14	1.84	0.08	2616.5	0.058
Transitivity	0.56	0.08	0.53	0.08	2739.0	0.014*
Diameter	2.02	0.55	1.98	0.56	2206.0	0.966

(CORR as the connectivity method in Alpha-1 frequency band, the rank-sum test was used to compare the difference as normality was not satisfied, * $P<0.05$)

Table S2-13: Univariate analysis of static network characteristic

Item	P_{50}_PC	*IQR*_PC	P_{50}_PE	*IQR*_PE	*W*	*P*
Little world index	1.27	0.30	1.29	0.27	2250.0	0.809
Average vertex strength	0.60	0.23	0.95	0.49	843.0	<0.001*
Average path length	1.64	0.05	1.63	0.05	2528.5	0.133
Transitivity	0.45	0.07	0.45	0.06	2351.0	0.485
Diameter	0.22	0.10	0.35	0.20	926.5	<0.001*

(PLI as the connectivity method in Alpha-2 frequency band, the rank-sum test was used to compare the difference as normality was not satisfied, * P<0.05)

Table S2-14: Univariate analysis of static network characteristic

Item	P_{50}_PC	*IQR*_PC	P_{50}_PE	*IQR*_PE	*W*	*P*
Little world index	1.05	0.14	1.03	0.18	2213.0	0.941
Average vertex strength	3.96	1.03	4.41	0.80	1783.0	0.063
Average path length	1.82	0.13	1.84	0.13	2005.0	0.390
Transitivity	0.56	0.15	0.58	0.11	2105.0	0.683
Diameter	1.07	0.76	1.24	0.62	1893.0	0.172

(MSC as the connectivity method in Alpha-2 frequency band, the rank-sum test was used to compare the difference as normality was not satisfied, * P<0.05)

Table S2-15: Univariate analysis of static network characteristic

Item	P_{50}_PC	*IQR*_PC	P_{50}_PE	*IQR*_PE	*W*	*P*
Little world index	1.16	0.21	1.15	0.25	2466.0	0.224
Average vertex strength	1.04	0.74	1.54	0.95	1255.0	<0.001*
Average path length	1.57	0.03	1.57	0.04	2356.0	0.470
Transitivity	0.47	0.06	0.49	0.06	1794.0	0.070*
Diameter	0.31	0.26	0.46	0.28	1309.0	<0.001*

(iCOH as the connectivity method in Alpha-2 frequency band, the rank-sum test was used to compare the difference as normality was not satisfied, * P<0.05)

Table S2-16: Univariate analysis of static network characteristic

Item	P_{50}_PC	*IQR*_PC	P_{50}_PE	*IQR*_PE	*W*	*P*
Little world index	0.98	0.14	0.98	0.15	1893.0	0.172
Average vertex strength	5.33	0.21	5.59	0.40	1051.0	<0.001*
Average path length	1.85	0.14	1.84	0.10	2481.0	0.199
Transitivity	0.55	0.08	0.52	0.08	2690.0	0.026*
Diameter	2.01	0.56	2.05	0.55	2105.0	0.683

(CORR as the connectivity method in Alpha-2 frequency band, the rank-sum test was used to compare the difference as normality was not satisfied, * P<0.05)

Table S2-17: Univariate analysis of static network characteristic

Item	P_{50}_PC	IQR_PC	P_{50}_PE	IQR_PE	W	P
Little world index	1.27	0.25	1.30	0.33	2129.0	0.764
Average vertex strength	0.57	0.22	0.90	0.47	774.0	<0.001*
Average path length	1.64	0.05	1.63	0.05	2391.0	0.379
Transitivity	0.46	0.06	0.46	0.07	2092.0	0.640
Diameter	0.20	0.09	0.34	0.21	818.0	<0.001*

(PLI as the connectivity method in Beta frequency band, the rank-sum test was used to compare the difference as normality was not satisfied, * $P<0.05$)

Table S2-18: Univariate analysis of static network characteristic

Item	P_{50}_PC	IQR_PC	P_{50}_PE	IQR_PE	W	P
Little world index	0.95	0.11	1.02	0.21	1443.0	0.001*
Average vertex strength	3.31	0.31	3.55	0.96	1224.0	<0.001*
Average path length	1.82	0.10	1.84	0.10	1811.5	0.083
Transitivity	0.51	0.07	0.54	0.10	1757.5	0.048*
Diameter	0.63	0.21	0.94	0.61	1307.0	<0.001*

(MSC as the connectivity method in Beta frequency band, the rank-sum test was used to compare the difference as normality was not satisfied, * $P<0.05$)

Table S2-19: Univariate analysis of static network characteristic

Item	P_{50}_PC	IQR_PC	P_{50}_PE	IQR_PE	W	P
Little world index	1.12	0.17	1.12	0.29	1935.0	0.239
Average vertex strength	0.31	0.14	0.80	0.56	194.0	<0.001*
Average path length	1.57	0.03	1.57	0.04	2355.5	0.471
Transitivity	0.47	0.05	0.48	0.07	1848.5	0.117
Diameter	0.09	0.05	0.23	0.17	255.0	<0.001*

(iCOH as the connectivity method in Beta frequency band, the rank-sum test was used to compare the difference as normality was not satisfied, * $P<0.05$)

Table S2-20: Univariate analysis of static network characteristic

Item	P_{50}_PC	IQR_PC	P_{50}_PE	IQR_PE	W	P
Little world index	0.97	0.15	0.99	0.17	1720.0	0.032*
Average vertex strength	5.29	0.20	5.56	0.39	978.0	<0.001*
Average path length	1.86	0.15	1.84	0.10	2473.0	0.212
Transitivity	0.55	0.09	0.53	0.07	2563.0	0.098
Diameter	1.98	0.54	2.01	0.51	1965.0	0.298

(CORR as the connectivity method in Beta frequency band, the rank-sum test was used to compare the difference as normality was not satisfied, * $P<0.05$)

Table S2-21: Univariate analysis of static network characteristic

Item	P_{50}_PC	*IQR*_PC	P_{50}_PE	*IQR*_PE	*W*	*P*
Little world index	1.28	0.24	1.31	0.28	2022.0	0.433
Average vertex strength	0.57	0.22	0.90	0.47	777.0	<0.001*
Average path length	1.64	0.05	1.62	0.06	2589.5	0.076
Transitivity	0.46	0.06	0.46	0.08	2036.0	0.471
Diameter	0.20	0.08	0.34	0.22	795.5	<0.001*

(PLI as the connectivity method in full frequency band, the rank-sum test was used to compare the difference as normality was not satisfied, * $P<0.05$)

Table S2-22: Univariate analysis of static network characteristic

Item	P_{50}_PC	*IQR*_PC	P_{50}_PE	*IQR*_PE	*W*	*P*
Little world index	0.97	0.10	1.00	0.16	1807.0	0.079
Average vertex strength	3.41	0.27	3.63	0.56	1200.0	<0.001*
Average path length	1.82	0.13	1.82	0.06	2180.5	0.946
Transitivity	0.52	0.08	0.51	0.09	2437.0	0.277
Diameter	0.67	0.22	0.85	0.34	1358.0	<0.001*

(MSC as the connectivity method in full frequency band, the rank-sum test was used to compare the difference as normality was not satisfied, * $P<0.05$)

Table S2-23: Univariate analysis of static network characteristic

Item	P_{50}_PC	*IQR*_PC	P_{50}_PE	*IQR*_PE	*W*	*P*
Little world index	1.06	0.17	1.11	0.19	1715.0	0.030*
Average vertex strength	0.26	0.11	0.57	0.38	246.0	<0.001*
Average path length	1.57	0.02	1.58	0.03	1456.5	0.001*
Transitivity	0.45	0.06	0.47	0.06	1697.5	0.025*
Diameter	0.08	0.04	0.17	0.12	229.0	<0.001*

(iCOH as the connectivity method in full frequency band, the rank-sum test was used to compare the difference as normality was not satisfied, * $P<0.05$)

Table S2-24: Univariate analysis of static network characteristic

Item	P_{50}_PC	*IQR*_PC	P_{50}_PE	*IQR*_PE	*W*	*P*
Little world index	0.97	0.16	0.99	0.17	1777.0	0.059
Average vertex strength	5.28	0.19	5.56	0.39	976.0	<0.001*
Average path length	1.85	0.15	1.84	0.10	2488.5	0.187
Transitivity	0.55	0.08	0.53	0.07	2580.0	0.083
Diameter	1.98	0.54	2.01	0.51	1942.0	0.252

(CORR as the connectivity method in full frequency band, the rank-sum test was used to compare the difference as normality was not satisfied, * $P<0.05$)

Supplementary Part S3

Univariate analysis of dynamic network characteristics-Original sequence

EEG signals dataset

Table S3-1: Univariate analysis of dynamic network characteristic

Item	P_{50}_PC	*IQR*_PC	P_{50}_PE	*IQR*_PE	*W*	*P*
Mean-Little world index	1.13	0.03	1.16	0.05	84.0	0.015*
**Standard Deviation*-Little world index	0.18	0.02	0.18	0.01	117.0	0.178
P_{50}-Little world index	1.12	0.05	1.14	0.04	83.0	0.014*
IQR-Little world index	0.24	0.05	0.25	0.05	132.0	0.386
Mean-Average vertex strength	2.60	0.23	2.91	0.33	65.0	0.002*
Standard Deviation-Average vertex strength	0.38	0.12	0.49	0.22	53.0	<0.001*
P_{50}-Average vertex strength	2.59	0.26	2.82	0.17	63.0	0.002*
IQR-Average vertex strength	0.51	0.18	0.66	0.30	43.0	<0.001*
Mean-Average path length	1.64	0.01	1.64	0.01	75.0	0.006*
Standard Deviation-Average path length	0.03	0.01	0.04	0.01	84.0	0.015*
P_{50}-Average path length	1.63	0.01	1.64	0.01	44.0	<0.001*
IQR-Average path length	0.04	0.01	0.05	0.02	86.5	0.019*
Mean-Transitivity	0.44	0.01	0.45	0.02	60.0	0.001*
Standard Deviation-Transitivity	0.04	0.01	0.05	0.00	55.0	0.001*
P_{50}-Transitivity	0.44	0.01	0.45	0.02	62.5	0.002*
IQR-Transitivity	0.06	0.01	0.07	0.01	71.0	0.004*
Mean-Diameter	0.96	0.09	1.09	0.14	57.0	0.001*
Standard Deviation-Diameter	0.19	0.05	0.26	0.10	60.0	0.001*
P_{50}-Diameter	0.94	0.08	1.04	0.12	55.5	0.001*
IQR-Diameter	0.23	0.08	0.32	0.11	44.0	<0.001*

(PLI as the connectivity method in Delta frequency band, the rank-sum test was used to compare the difference as normality was not satisfied, * $P<0.05$)

Table S3-2: Univariate analysis of dynamic network characteristic

Item	P_{50}_PC	*IQR*_PC	P_{50}_PE	*IQR*_PE	*W*	*P*
Mean-Little world index	1.06	0.03	1.09	0.03	77.0	0.007*
Standard Deviation-Little world index	0.14	0.02	0.14	0.02	134.0	0.422
P_{50}-Little world index	1.05	0.03	1.09	0.03	72.0	0.004*
IQR-Little world index	0.16	0.04	0.19	0.03	89.0	0.023*
Mean-Average vertex strength	5.13	0.26	5.45	0.46	13.0	<0.001*
Standard Deviation-Average vertex strength	0.42	0.08	0.44	0.10	126.0	0.290
P_{50}-Average vertex strength	5.09	0.26	5.46	0.52	14.0	<0.001*
IQR-Average vertex strength	0.56	0.11	0.62	0.13	100.0	0.058
Mean-Average path length	1.87	0.02	1.87	0.03	137.0	0.479
Standard Deviation-Average path length	0.14	0.05	0.16	0.03	125.0	0.276
P_{50}-Average path length	1.86	0.02	1.86	0.03	122.0	0.231
IQR-Average path length	0.14	0.05	0.17	0.05	82.0	0.014*
Mean-Transitivity	0.58	0.03	0.61	0.04	49.0	<0.001*
Standard Deviation-Transitivity	0.08	0.01	0.08	0.01	157.0	0.937
P_{50}-Transitivity	0.57	0.04	0.61	0.05	56.0	0.001*
IQR-Transitivity	0.11	0.04	0.10	0.02	164.0	0.912
Mean-Diameter	1.86	0.17	2.14	0.28	13.0	<0.001*
Standard Deviation-Diameter	0.48	0.08	0.52	0.08	81.0	0.011*
P_{50}-Diameter	1.78	0.17	2.06	0.31	14.0	<0.001*
IQR-Diameter	0.58	0.14	0.63	0.24	116.0	0.168

(MSC as the connectivity method in Delta frequency band, the rank-sum test was used to compare the difference as normality was not satisfied, * P<0.05)

Table S3-3: Univariate analysis of dynamic network characteristic

Item	P_{50}_PC	*IQR*_PC	P_{50}_PE	*IQR*_PE	*W*	*P*
Mean-Little world index	1.05	0.02	1.05	0.03	140.0	0.539
Standard Deviation-Little world index	0.14	0.02	0.17	0.02	18.0	<0.001*
P_{50}-Little world index	1.05	0.02	1.05	0.04	161.0	0.987
IQR-Little world index	0.17	0.03	0.22	0.03	30.0	<0.001*
Mean-Average vertex strength	2.01	0.11	2.15	0.19	91.0	0.028*
Standard Deviation-Average vertex strength	0.33	0.06	0.41	0.14	34.0	<0.001*
P_{50}-Average vertex strength	1.95	0.10	2.06	0.18	82.0	0.012*
IQR-Average vertex strength	0.45	0.11	0.58	0.11	32.0	<0.001*
Mean-Average path length	1.58	0.00	1.58	0.00	116.0	0.168
Standard Deviation-Average path length	0.02	0.01	0.03	0.01	71.0	0.004*
P_{50}-Average path length	1.57	0.00	1.57	0.01	139.5	0.481
IQR-Average path length	0.03	0.01	0.03	0.01	68.5	0.002*
Mean-Transitivity	0.45	0.01	0.45	0.01	196.0	0.262
Standard Deviation-Transitivity	0.04	0.01	0.05	0.01	53.0	<0.001*
P_{50}-Transitivity	0.45	0.01	0.45	0.01	131.5	0.373
IQR-Transitivity	0.06	0.01	0.07	0.01	93.0	0.033*
Mean-Diameter	0.63	0.04	0.66	0.06	78.0	0.008*
Standard Deviation-Diameter	0.13	0.02	0.16	0.04	23.0	<0.001*
P_{50}-Diameter	0.61	0.04	0.64	0.05	86.0	0.018*
IQR-Diameter	0.16	0.04	0.22	0.06	35.0	<0.001*

(iCOH as the connectivity method in Delta frequency band, the rank-sum test was used to compare the difference as normality was not satisfied, * $P<0.05$)

Table S3-4: Univariate analysis of dynamic network characteristic

Item	P_{50}_PC	IQR_PC	P_{50}_PE	IQR_PE	W	P
Mean-Little world index	1.03	0.04	1.09	0.05	76.0	0.007*
Standard Deviation-Little world index	0.14	0.04	0.13	0.03	148.0	0.718
P_{50}-Little world index	1.01	0.04	1.07	0.06	71.0	0.004*
IQR-Little world index	0.16	0.04	0.17	0.03	139.0	0.519
Mean-Average vertex strength	5.75	0.29	6.07	0.33	26.0	<0.001*
Standard Deviation-Average vertex strength	0.34	0.09	0.33	0.08	170.0	0.765
P_{50}-Average vertex strength	5.75	0.29	6.07	0.36	20.0	<0.001*
IQR-Average vertex strength	0.44	0.17	0.41	0.13	172.0	0.718
Mean-Average path length	1.85	0.03	1.87	0.03	104.0	0.077
Standard Deviation-Average path length	0.13	0.04	0.16	0.03	100.0	0.058
P_{50}-Average path length	1.85	0.03	1.87	0.02	105.5	0.085
IQR-Average path length	0.12	0.03	0.15	0.05	103.0	0.072
Mean-Transitivity	0.57	0.04	0.62	0.05	84.0	0.015*
Standard Deviation-Transitivity	0.07	0.02	0.08	0.01	155.0	0.888
P_{50}-Transitivity	0.57	0.04	0.61	0.05	86.5	0.020*
IQR-Transitivity	0.10	0.03	0.10	0.02	131.0	0.369
Mean-Diameter	2.31	0.31	2.54	0.29	49.0	<0.001*
Standard Deviation-Diameter	0.49	0.10	0.55	0.11	95.0	0.039*
P_{50}-Diameter	2.25	0.23	2.47	0.36	48.0	<0.001*
IQR-Diameter	0.60	0.20	0.69	0.22	111.0	0.124

(CORR as the connectivity method in Delta frequency band, the rank-sum test was used to compare the difference as normality was not satisfied, * $P<0.05$)

Table S3-5: Univariate analysis of dynamic network characteristic

Item	P_{50}_PC	*IQR*_PC	P_{50}_PE	*IQR*_PE	*W*	*P*
Mean-Little world index	1.13	0.03	1.16	0.05	80.0	0.010*
Standard Deviation-Little world index	0.17	0.02	0.18	0.02	108.0	0.102
P_{50}-Little world index	1.12	0.02	1.16	0.06	83.0	0.014*
IQR-Little world index	0.23	0.05	0.24	0.02	101.0	0.062
Mean-Average vertex strength	2.19	0.34	2.48	0.40	60.0	0.001*
Standard Deviation-Average vertex strength	0.32	0.14	0.46	0.20	56.0	0.001*
P_{50}-Average vertex strength	2.11	0.33	2.45	0.33	59.0	0.001*
IQR-Average vertex strength	0.42	0.13	0.61	0.30	40.0	<0.001*
Mean-Average path length	1.64	0.01	1.64	0.01	144.0	0.626
Standard Deviation-Average path length	0.04	0.01	0.04	0.01	105.0	0.083
P_{50}-Average path length	1.64	0.01	1.64	0.01	127.0	0.278
IQR-Average path length	0.04	0.01	0.05	0.02	91.0	0.024*
Mean-Transitivity	0.44	0.01	0.45	0.02	80.0	0.010*
Standard Deviation-Transitivity	0.04	0.01	0.05	0.01	91.0	0.028*
P_{50}-Transitivity	0.44	0.01	0.45	0.02	77.0	0.007*
IQR-Transitivity	0.06	0.01	0.06	0.01	101.0	0.062
Mean-Diameter	0.80	0.15	0.92	0.15	62.0	0.001*
Standard Deviation-Diameter	0.16	0.08	0.23	0.10	61.0	0.001*
P_{50}-Diameter	0.77	0.13	0.90	0.15	64.0	0.002*
IQR-Diameter	0.20	0.07	0.28	0.14	51.0	0.001*

(PLI as the connectivity method in Theta frequency band, the rank-sum test was used to compare the difference as normality was not satisfied, * $P<0.05$)

Table S3-6: Univariate analysis of dynamic network characteristic

Item	P_{50}_PC	IQR_PC	P_{50}_PE	IQR_PE	W	P
Mean-Little world index	1.03	0.03	1.08	0.06	40.0	<0.001*
Standard Deviation-Little world index	0.12	0.02	0.14	0.03	69.0	0.003*
P_{50}-Little world index	1.03	0.04	1.07	0.07	54.0	<0.001*
IQR-Little world index	0.16	0.04	0.17	0.02	106.0	0.089
Mean-Average vertex strength	4.93	0.33	5.60	0.43	18.0	<0.001*
Standard Deviation-Average vertex strength	0.32	0.11	0.44	0.07	56.0	0.001*
P_{50}-Average vertex strength	4.90	0.28	5.56	0.38	17.0	<0.001*
IQR-Average vertex strength	0.43	0.12	0.58	0.06	37.0	<0.001*
Mean-Average path length	1.86	0.03	1.87	0.05	126.0	0.290
Standard Deviation-Average path length	0.11	0.04	0.14	0.04	66.0	0.002*
P_{50}-Average path length	1.86	0.02	1.88	0.04	79.5	0.011*
IQR-Average path length	0.12	0.03	0.15	0.05	65.0	0.003*
Mean-Transitivity	0.55	0.03	0.62	0.03	31.0	<0.001*
Standard Deviation-Transitivity	0.07	0.02	0.08	0.01	124.0	0.262
P_{50}-Transitivity	0.55	0.03	0.62	0.04	35.0	<0.001*
IQR-Transitivity	0.09	0.02	0.10	0.01	127.0	0.305
Mean-Diameter	1.72	0.22	2.25	0.25	19.0	<0.001*
Standard Deviation-Diameter	0.38	0.08	0.51	0.11	51.0	<0.001*
P_{50}-Diameter	1.67	0.19	2.16	0.23	15.0	<0.001*
IQR-Diameter	0.48	0.14	0.65	0.12	34.0	<0.001*

(MSC as the connectivity method in Theta frequency band, the rank-sum test was used to compare the difference as normality was not satisfied, * P<0.05)

Table S3-7: Univariate analysis of dynamic network characteristic

Item	P_{50}_PC	*IQR*_PC	P_{50}_PE	*IQR*_PE	*W*	*P*
Mean-Little world index	1.07	0.02	1.07	0.04	154.0	0.863
Standard Deviation-Little world index	0.13	0.01	0.17	0.02	36.0	<0.001*
P_{50}-Little world index	1.07	0.02	1.06	0.04	165.0	0.888
IQR-Little world index	0.15	0.02	0.19	0.03	35.0	<0.001*
Mean-Average vertex strength	1.98	0.12	2.27	0.36	36.0	<0.001*
Standard Deviation-Average vertex strength	0.34	0.06	0.49	0.14	14.0	<0.001*
P_{50}-Average vertex strength	1.94	0.15	2.24	0.40	33.0	<0.001*
IQR-Average vertex strength	0.39	0.11	0.64	0.20	18.0	<0.001*
Mean-Average path length	1.58	0.00	1.58	0.00	98.0	0.049*
Standard Deviation-Average path length	0.02	0.00	0.03	0.01	21.0	<0.001*
P_{50}-Average path length	1.57	0.01	1.58	0.01	89.5	0.013*
IQR-Average path length	0.02	0.01	0.04	0.01	48.0	<0.001*
Mean-Transitivity	0.46	0.01	0.46	0.01	158.0	0.962
Standard Deviation-Transitivity	0.04	0.00	0.05	0.01	43.0	<0.001*
P_{50}-Transitivity	0.46	0.01	0.46	0.01	118.0	0.189
IQR-Transitivity	0.05	0.01	0.06	0.01	79.0	0.009*
Mean-Diameter	0.61	0.03	0.71	0.12	37.0	<0.001*
Standard Deviation-Diameter	0.13	0.02	0.19	0.06	8.0	<0.001*
P_{50}-Diameter	0.59	0.05	0.68	0.15	39.0	<0.001*
IQR-Diameter	0.15	0.03	0.21	0.07	19.0	<0.001*

(iCOH as the connectivity method in Theta frequency band, the rank-sum test was used to compare the difference as normality was not satisfied, * $P<0.05$)

Table S3-8: Univariate analysis of dynamic network characteristic

Item	P_{50}_PC	IQR_PC	P_{50}_PE	IQR_PE	W	P
Mean-Little world index	1.02	0.06	1.07	0.07	76.0	0.007*
Standard Deviation-Little world index	0.13	0.04	0.12	0.02	167.0	0.838
P_{50}-Little world index	1.00	0.07	1.06	0.08	69.0	0.003*
IQR-Little world index	0.15	0.06	0.16	0.03	143.0	0.604
Mean-Average vertex strength	5.62	0.25	6.04	0.33	19.0	<0.001*
Standard Deviation-Average vertex strength	0.31	0.10	0.30	0.10	162.0	0.962
P_{50}-Average vertex strength	5.61	0.25	6.07	0.29	18.0	<0.001*
IQR-Average vertex strength	0.39	0.14	0.38	0.17	135.0	0.440
Mean-Average path length	1.85	0.05	1.86	0.06	107.0	0.095
Standard Deviation-Average path length	0.11	0.07	0.14	0.06	118.0	0.189
P_{50}-Average path length	1.85	0.03	1.88	0.05	94.0	0.037*
IQR-Average path length	0.13	0.05	0.14	0.05	126.5	0.293
Mean-Transitivity	0.57	0.05	0.62	0.05	79.0	0.009*
Standard Deviation-Transitivity	0.07	0.02	0.07	0.01	152.0	0.814
P_{50}-Transitivity	0.57	0.05	0.61	0.05	86.5	0.020*
IQR-Transitivity	0.10	0.03	0.10	0.03	156.0	0.912
Mean-Diameter	2.18	0.25	2.55	0.27	42.0	<0.001*
Standard Deviation-Diameter	0.44	0.09	0.52	0.18	93.0	0.033*
P_{50}-Diameter	2.16	0.21	2.46	0.23	43.0	<0.001*
IQR-Diameter	0.54	0.11	0.65	0.24	102.0	0.067

(CORR as the connectivity method in Theta frequency band, the rank-sum test was used to compare the difference as normality was not satisfied, * $P<0.05$)

Table S3-9: Univariate analysis of dynamic network characteristic

Item	P_{50}_PC	*IQR*_PC	P_{50}_PE	*IQR*_PE	*W*	*P*
Mean-Little world index	1.14	0.03	1.16	0.03	78.0	0.008*
Standard Deviation-Little world index	0.18	0.02	0.19	0.02	84.0	0.015*
P_{50}-Little world index	1.13	0.03	1.14	0.04	75.0	0.006*
IQR-Little world index	0.23	0.05	0.25	0.03	113.0	0.140
Mean-Average vertex strength	1.99	0.43	2.42	0.43	58.0	0.001*
Standard Deviation-Average vertex strength	0.30	0.12	0.46	0.21	52.0	<0.001*
P_{50}-Average vertex strength	1.91	0.40	2.39	0.34	53.5	0.001*
IQR-Average vertex strength	0.40	0.10	0.63	0.31	38.0	<0.001*
Mean-Average path length	1.64	0.01	1.64	0.01	118.0	0.189
Standard Deviation-Average path length	0.04	0.01	0.04	0.02	81.0	0.011*
P_{50}-Average path length	1.63	0.01	1.64	0.01	99.0	0.045*
IQR-Average path length	0.04	0.01	0.05	0.01	89.0	0.023*
Mean-Transitivity	0.44	0.01	0.45	0.02	60.0	0.001*
Standard Deviation-Transitivity	0.05	0.01	0.05	0.01	76.0	0.007*
P_{50}-Transitivity	0.44	0.01	0.45	0.02	59.5	0.001*
IQR-Transitivity	0.06	0.01	0.07	0.01	84.0	0.015*
Mean-Diameter	0.74	0.16	0.90	0.17	52.0	<0.001*
Standard Deviation-Diameter	0.15	0.05	0.24	0.11	40.0	<0.001*
P_{50}-Diameter	0.69	0.16	0.88	0.17	48.0	<0.001*
IQR-Diameter	0.17	0.06	0.28	0.15	38.5	<0.001*

(PLI as the connectivity method in Alpha-1 frequency band, the rank-sum test was used to compare the difference as normality was not satisfied, * $P<0.05$)

Table S3-10: Univariate analysis of dynamic network characteristic

Item	P_{50}_PC	IQR_PC	P_{50}_PE	IQR_PE	W	P
Mean-Little world index	1.05	0.04	1.12	0.04	49.0	<0.001*
Standard Deviation-Little world index	0.12	0.02	0.16	0.03	59.0	0.001*
P_{50}-Little world index	1.05	0.04	1.10	0.04	59.0	0.001*
IQR-Little world index	0.15	0.04	0.19	0.03	88.0	0.021*
Mean-Average vertex strength	5.14	0.58	5.69	0.53	49.0	<0.001*
Standard Deviation-Average vertex strength	0.38	0.14	0.57	0.11	35.0	<0.001*
P_{50}-Average vertex strength	5.10	0.55	5.67	0.61	54.0	<0.001*
IQR-Average vertex strength	0.52	0.20	0.78	0.20	43.0	<0.001*
Mean-Average path length	1.87	0.02	1.85	0.03	225.0	0.039*
Standard Deviation-Average path length	0.12	0.06	0.18	0.03	49.0	<0.001*
P_{50}-Average path length	1.87	0.04	1.87	0.03	165.5	0.873
IQR-Average path length	0.12	0.06	0.19	0.06	44.0	<0.001*
Mean-Transitivity	0.57	0.07	0.63	0.03	68.0	0.003*
Standard Deviation-Transitivity	0.07	0.02	0.08	0.02	106.0	0.089
P_{50}-Transitivity	0.57	0.08	0.63	0.04	72.0	0.005*
IQR-Transitivity	0.09	0.03	0.10	0.02	103.0	0.072
Mean-Diameter	1.87	0.48	2.22	0.33	62.0	0.001*
Standard Deviation-Diameter	0.44	0.19	0.58	0.07	57.0	0.001*
P_{50}-Diameter	1.81	0.48	2.13	0.35	65.0	0.002*
IQR-Diameter	0.56	0.22	0.72	0.17	69.0	0.003*

(MSC as the connectivity method in Alpha-1 frequency band, the rank-sum test was used to compare the difference as normality was not satisfied, * $P<0.05$)

Table S3-11: Univariate analysis of dynamic network characteristic

Item	P_{50}_PC	IQR_PC	P_{50}_PE	IQR_PE	W	P
Mean-Little world index	1.05	0.02	1.04	0.04	178.0	0.582
Standard Deviation-Little world index	0.13	0.03	0.16	0.02	49.0	<0.001*
P_{50}-Little world index	1.05	0.04	1.03	0.05	176.0	0.626
IQR-Little world index	0.18	0.02	0.20	0.07	120.0	0.211
Mean-Average vertex strength	2.48	0.09	2.85	0.39	30.0	<0.001*
Standard Deviation-Average vertex strength	0.40	0.08	0.59	0.13	5.0	<0.001*
P_{50}-Average vertex strength	2.44	0.13	2.74	0.37	40.0	<0.001*
IQR-Average vertex strength	0.52	0.17	0.83	0.14	19.0	<0.001*
Mean-Average path length	1.58	0.00	1.58	0.01	147.0	0.694
Standard Deviation-Average path length	0.03	0.01	0.03	0.01	34.0	<0.001*
P_{50}-Average path length	1.57	0.00	1.58	0.01	104.5	0.058
IQR-Average path length	0.02	0.01	0.03	0.01	62.5	0.002*
Mean-Transitivity	0.45	0.01	0.45	0.01	181.0	0.519
Standard Deviation-Transitivity	0.04	0.01	0.05	0.01	62.0	0.001*
P_{50}-Transitivity	0.45	0.01	0.45	0.01	176.5	0.610
IQR-Transitivity	0.06	0.01	0.06	0.01	107.0	0.095
Mean-Diameter	0.76	0.04	0.89	0.12	32.0	<0.001*
Standard Deviation-Diameter	0.15	0.04	0.23	0.04	19.0	<0.001*
P_{50}-Diameter	0.74	0.05	0.83	0.13	44.0	<0.001*
IQR-Diameter	0.20	0.06	0.31	0.08	24.0	<0.001*

(iCOH as the connectivity method in Alpha-1 frequency band, the rank-sum test was used to compare the difference as normality was not satisfied, * P<0.05)

Table S3-12: Univariate analysis of dynamic network characteristic

Item	P_{50}_PC	IQR_PC	P_{50}_PE	IQR_PE	W	P
Mean-Little world index	1.01	0.06	1.06	0.07	66.0	0.002*
Standard Deviation-Little world index	0.13	0.04	0.13	0.03	137.0	0.479
P_{50}-Little world index	1.00	0.05	1.06	0.07	62.0	0.001*
IQR-Little world index	0.14	0.06	0.16	0.04	124.0	0.262
Mean-Average vertex strength	5.59	0.22	6.03	0.31	17.0	<0.001*
Standard Deviation-Average vertex strength	0.30	0.10	0.30	0.11	153.0	0.838
P_{50}-Average vertex strength	5.54	0.23	6.05	0.28	18.0	<0.001*
IQR-Average vertex strength	0.38	0.15	0.38	0.20	143.0	0.604
Mean-Average path length	1.86	0.04	1.86	0.06	122.0	0.236
Standard Deviation-Average path length	0.12	0.05	0.13	0.06	111.0	0.124
P_{50}-Average path length	1.86	0.04	1.87	0.05	100.5	0.060
IQR-Average path length	0.12	0.05	0.14	0.05	128.5	0.323
Mean-Transitivity	0.56	0.05	0.61	0.05	78.0	0.008*
Standard Deviation-Transitivity	0.07	0.02	0.07	0.01	127.0	0.305
P_{50}-Transitivity	0.57	0.04	0.61	0.06	87.5	0.022*
IQR-Transitivity	0.10	0.02	0.10	0.03	144.0	0.626
Mean-Diameter	2.18	0.24	2.55	0.24	45.0	<0.001*
Standard Deviation-Diameter	0.44	0.13	0.54	0.17	94.0	0.036*
P_{50}-Diameter	2.13	0.21	2.44	0.22	43.0	<0.001*
IQR-Diameter	0.55	0.14	0.61	0.20	104.0	0.077

(CORR as the connectivity method in Alpha-1 frequency band, the rank-sum test was used to compare the difference as normality was not satisfied, * P<0.05)

Table S3-13: Univariate analysis of dynamic network characteristic

Item	P_{50}_PC	IQR_PC	P_{50}_PE	IQR_PE	W	P
Mean-Little world index	1.15	0.04	1.17	0.05	83.0	0.014*
Standard Deviation-Little world index	0.17	0.03	0.19	0.02	64.0	0.002*
P_{50}-Little world index	1.14	0.04	1.15	0.05	107.0	0.095
IQR-Little world index	0.22	0.04	0.26	0.05	99.0	0.053
Mean-Average vertex strength	1.90	0.47	2.39	0.44	56.0	0.001*
Standard Deviation-Average vertex strength	0.30	0.13	0.47	0.22	53.0	<0.001*
P_{50}-Average vertex strength	1.81	0.42	2.33	0.35	54.0	0.001*
IQR-Average vertex strength	0.39	0.12	0.61	0.34	42.0	<0.001*
Mean-Average path length	1.64	0.01	1.64	0.01	123.0	0.245
Standard Deviation-Average path length	0.04	0.01	0.04	0.02	110.0	0.116
P_{50}-Average path length	1.63	0.01	1.64	0.01	113.0	0.125
IQR-Average path length	0.04	0.01	0.04	0.02	143.0	0.596
Mean-Transitivity	0.44	0.01	0.45	0.02	72.0	0.004*
Standard Deviation-Transitivity	0.05	0.00	0.05	0.01	71.0	0.004*
P_{50}-Transitivity	0.44	0.01	0.45	0.02	67.5	0.003*
IQR-Transitivity	0.06	0.01	0.07	0.01	65.0	0.002*
Mean-Diameter	0.71	0.18	0.88	0.17	55.0	0.001*
Standard Deviation-Diameter	0.15	0.06	0.23	0.11	48.0	<0.001*
P_{50}-Diameter	0.67	0.17	0.87	0.17	51.0	0.001*
IQR-Diameter	0.18	0.07	0.28	0.13	38.5	<0.001*

(PLI as the connectivity method in Alpha-2 frequency band, the rank-sum test was used to compare the difference as normality was not satisfied, * $P<0.05$)

Table S3-14: Univariate analysis of dynamic network characteristic

Item	P_{50}_PC	IQR_PC	P_{50}_PE	IQR_PE	W	P
Mean-Little world index	1.05	0.07	1.11	0.06	63.0	0.002*
Standard Deviation-Little world index	0.13	0.03	0.16	0.03	79.0	0.009*
P_{50}-Little world index	1.04	0.07	1.10	0.05	62.0	0.001*
IQR-Little world index	0.16	0.02	0.19	0.06	78.0	0.008*
Mean-Average vertex strength	5.09	0.82	5.76	0.47	68.0	0.003*
Standard Deviation-Average vertex strength	0.42	0.19	0.58	0.16	57.0	0.001*
P_{50}-Average vertex strength	5.06	0.83	5.70	0.49	77.0	0.007*
IQR-Average vertex strength	0.50	0.24	0.74	0.31	59.0	0.001*
Mean-Average path length	1.86	0.03	1.85	0.07	187.0	0.404
Standard Deviation-Average path length	0.13	0.07	0.18	0.05	66.0	0.002*
P_{50}-Average path length	1.86	0.03	1.86	0.04	163.0	0.936
IQR-Average path length	0.13	0.08	0.18	0.07	66.0	0.003*
Mean-Transitivity	0.57	0.07	0.63	0.03	72.0	0.004*
Standard Deviation-Transitivity	0.07	0.01	0.08	0.02	92.0	0.030*
P_{50}-Transitivity	0.56	0.08	0.62	0.04	71.0	0.004*
IQR-Transitivity	0.09	0.03	0.11	0.04	117.0	0.178
Mean-Diameter	1.84	0.61	2.23	0.29	79.0	0.009*
Standard Deviation-Diameter	0.44	0.15	0.57	0.09	70.0	0.003*
P_{50}-Diameter	1.76	0.63	2.16	0.29	83.0	0.014*
IQR-Diameter	0.55	0.28	0.73	0.16	96.0	0.042*

(MSC as the connectivity method in Alpha-2 frequency band, the rank-sum test was used to compare the difference as normality was not satisfied, * P<0.05)

Table S3-15: Univariate analysis of dynamic network characteristic

Item	P_{50}_PC	IQR_PC	P_{50}_PE	IQR_PE	W	P
Mean-Little world index	1.05	0.05	1.04	0.04	192.0	0.320
Standard Deviation-Little world index	0.13	0.04	0.16	0.02	88.0	0.021*
P_{50}-Little world index	1.04	0.04	1.05	0.03	173.0	0.694
IQR-Little world index	0.17	0.05	0.19	0.04	88.0	0.021*
Mean-Average vertex strength	2.53	0.26	2.84	0.46	47.0	<0.001*
Standard Deviation-Average vertex strength	0.45	0.17	0.58	0.19	29.0	<0.001*
P_{50}-Average vertex strength	2.53	0.24	2.75	0.57	58.0	0.001*
IQR-Average vertex strength	0.51	0.21	0.76	0.21	49.0	<0.001*
Mean-Average path length	1.58	0.00	1.58	0.01	132.0	0.386
Standard Deviation-Average path length	0.02	0.01	0.04	0.01	53.0	<0.001*
P_{50}-Average path length	1.57	0.00	1.58	0.01	114.5	0.117
IQR-Average path length	0.02	0.01	0.04	0.01	81.5	0.011*
Mean-Transitivity	0.45	0.01	0.45	0.01	172.0	0.718
Standard Deviation-Transitivity	0.04	0.01	0.05	0.01	75.0	0.006*
P_{50}-Transitivity	0.45	0.01	0.45	0.01	166.5	0.849
IQR-Transitivity	0.06	0.01	0.06	0.02	111.0	0.124
Mean-Diameter	0.79	0.07	0.89	0.17	48.0	<0.001*
Standard Deviation-Diameter	0.17	0.05	0.23	0.06	33.0	<0.001*
P_{50}-Diameter	0.76	0.08	0.85	0.21	59.0	0.001*
IQR-Diameter	0.20	0.08	0.30	0.07	35.0	<0.001*

(iCOH as the connectivity method in Alpha-2 frequency band, the rank-sum test was used to compare the difference as normality was not satisfied, * P<0.05)

Table S3-16: Univariate analysis of dynamic network characteristic

Item	P_{50}_PC	IQR_PC	P_{50}_PE	IQR_PE	W	P
Mean-Little world index	1.02	0.06	1.06	0.07	73.0	0.005*
Standard Deviation-Little world index	0.12	0.03	0.13	0.03	125.0	0.276
P_{50}-Little world index	1.01	0.05	1.05	0.07	70.0	0.003*
IQR-Little world index	0.15	0.06	0.16	0.03	126.0	0.290
Mean-Average vertex strength	5.56	0.23	6.02	0.30	17.0	<0.001*
Standard Deviation-Average vertex strength	0.29	0.10	0.30	0.11	148.0	0.718
P_{50}-Average vertex strength	5.54	0.23	6.03	0.30	18.0	<0.001*
IQR-Average vertex strength	0.38	0.15	0.38	0.19	129.0	0.336
Mean-Average path length	1.86	0.04	1.86	0.06	122.0	0.236
Standard Deviation-Average path length	0.11	0.05	0.13	0.06	115.0	0.158
P_{50}-Average path length	1.86	0.04	1.88	0.05	97.0	0.046*
IQR-Average path length	0.12	0.05	0.14	0.04	117.0	0.176
Mean-Transitivity	0.56	0.04	0.61	0.06	76.0	0.007*
Standard Deviation-Transitivity	0.07	0.02	0.07	0.01	134.0	0.422
P_{50}-Transitivity	0.56	0.05	0.61	0.06	85.0	0.016*
IQR-Transitivity	0.09	0.02	0.10	0.02	130.0	0.352
Mean-Diameter	2.16	0.25	2.53	0.31	42.0	<0.001*
Standard Deviation-Diameter	0.45	0.14	0.52	0.16	98.0	0.049*
P_{50}-Diameter	2.11	0.25	2.43	0.27	44.0	<0.001*
IQR-Diameter	0.57	0.18	0.62	0.15	121.0	0.223

(CORR as the connectivity method in Alpha-2 frequency band, the rank-sum test was used to compare the difference as normality was not satisfied, * P<0.05)

Table S3-17: Univariate analysis of dynamic network characteristic

Item	P_{50}_PC	IQR_PC	P_{50}_PE	IQR_PE	W	P
Mean-Little world index	1.13	0.03	1.16	0.02	48.0	<0.001*
Standard Deviation-Little world index	0.17	0.01	0.19	0.02	51.0	<0.001*
P_{50}-Little world index	1.12	0.02	1.16	0.05	49.0	<0.001*
IQR-Little world index	0.22	0.02	0.23	0.04	102.0	0.067
Mean-Average vertex strength	1.76	0.49	2.29	0.60	56.0	0.001*
Standard Deviation-Average vertex strength	0.29	0.13	0.47	0.23	52.0	<0.001*
P_{50}-Average vertex strength	1.66	0.43	2.28	0.54	57.0	0.001*
IQR-Average vertex strength	0.40	0.13	0.60	0.35	32.0	<0.001*
Mean-Average path length	1.64	0.01	1.64	0.01	104.0	0.077
Standard Deviation-Average path length	0.04	0.01	0.04	0.01	83.0	0.014*
P_{50}-Average path length	1.63	0.01	1.64	0.01	74.5	0.005*
IQR-Average path length	0.04	0.01	0.05	0.01	131.5	0.368
Mean-Transitivity	0.44	0.01	0.45	0.02	73.0	0.005*
Standard Deviation-Transitivity	0.05	0.00	0.05	0.01	65.0	0.002*
P_{50}-Transitivity	0.44	0.01	0.45	0.02	77.0	0.009*
IQR-Transitivity	0.06	0.01	0.06	0.01	113.0	0.140
Mean-Diameter	0.65	0.18	0.86	0.24	50.0	<0.001*
Standard Deviation-Diameter	0.14	0.05	0.23	0.12	51.0	<0.001*
P_{50}-Diameter	0.61	0.18	0.84	0.23	51.0	0.001*
IQR-Diameter	0.17	0.03	0.26	0.11	50.5	0.001*

(PLI as the connectivity method in Beta frequency band, the rank-sum test was used to compare the difference as normality was not satisfied, * P<0.05)

Table S3-18: Univariate analysis of dynamic network characteristic

Item	P_{50}_PC	*IQR*_PC	P_{50}_PE	*IQR*_PE	*W*	*P*
Mean-Little world index	0.92	0.06	1.03	0.13	36.0	<0.001*
Standard Deviation-Little world index	0.11	0.01	0.15	0.03	45.0	<0.001*
P_{50}-Little world index	0.92	0.06	1.01	0.12	49.0	<0.001*
IQR-Little world index	0.13	0.03	0.16	0.04	54.0	<0.001*
Mean-Average vertex strength	4.13	0.25	4.72	0.63	26.0	<0.001*
Standard Deviation-Average vertex strength	0.14	0.05	0.45	0.22	18.0	<0.001*
P_{50}-Average vertex strength	4.13	0.22	4.64	0.56	28.0	<0.001*
IQR-Average vertex strength	0.18	0.06	0.44	0.33	19.0	<0.001*
Mean-Average path length	1.82	0.03	1.82	0.06	146.0	0.671
Standard Deviation-Average path length	0.06	0.01	0.12	0.05	14.0	<0.001*
P_{50}-Average path length	1.81	0.02	1.81	0.04	137.5	0.480
IQR-Average path length	0.06	0.03	0.12	0.07	40.0	<0.001*
Mean-Transitivity	0.47	0.03	0.56	0.08	30.0	<0.001*
Standard Deviation-Transitivity	0.06	0.01	0.08	0.03	43.0	<0.001*
P_{50}-Transitivity	0.47	0.04	0.54	0.08	34.0	<0.001*
IQR-Transitivity	0.07	0.01	0.09	0.03	49.0	<0.001*
Mean-Diameter	1.17	0.18	1.56	0.36	25.0	<0.001*
Standard Deviation-Diameter	0.19	0.06	0.34	0.14	12.0	<0.001*
P_{50}-Diameter	1.17	0.17	1.48	0.28	30.0	<0.001*
IQR-Diameter	0.24	0.06	0.43	0.20	10.0	<0.001*

(MSC as the connectivity method in Beta frequency band, the rank-sum test was used to compare the difference as normality was not satisfied, * $P<0.05$)

Table S3-19: Univariate analysis of dynamic network characteristic

Item	P_{50}_PC	*IQR*_PC	P_{50}_PE	*IQR*_PE	*W*	*P*
Mean-Little world index	1.06	0.03	1.08	0.04	91.0	0.028*
Standard Deviation-Little world index	0.10	0.02	0.14	0.03	26.0	<0.001*
P_{50}-Little world index	1.06	0.02	1.09	0.05	78.0	0.008*
IQR-Little world index	0.13	0.02	0.18	0.04	41.0	<0.001*
Mean-Average vertex strength	1.12	0.05	1.59	0.38	1.0	<0.001*
Standard Deviation-Average vertex strength	0.14	0.04	0.38	0.14	3.0	<0.001*
P_{50}-Average vertex strength	1.10	0.04	1.55	0.36	1.0	<0.001*
IQR-Average vertex strength	0.17	0.05	0.52	0.21	3.0	<0.001*
Mean-Average path length	1.57	0.00	1.58	0.01	87.0	0.021*
Standard Deviation-Average path length	0.02	0.00	0.03	0.01	34.0	<0.001*
P_{50}-Average path length	1.57	0.01	1.57	0.00	108.5	0.083
IQR-Average path length	0.02	0.00	0.03	0.01	46.5	<0.001*
Mean-Transitivity	0.46	0.01	0.47	0.01	72.0	0.004*
Standard Deviation-Transitivity	0.03	0.00	0.04	0.01	33.0	<0.001*
P_{50}-Transitivity	0.46	0.01	0.47	0.01	58.0	0.001*
IQR-Transitivity	0.05	0.01	0.06	0.01	37.0	<0.001*
Mean-Diameter	0.34	0.01	0.49	0.12	1.0	<0.001*
Standard Deviation-Diameter	0.06	0.01	0.14	0.06	3.0	<0.001*
P_{50}-Diameter	0.33	0.02	0.47	0.11	2.0	<0.001*
IQR-Diameter	0.07	0.02	0.17	0.08	3.0	<0.001*

(iCOH as the connectivity method in Beta frequency band, the rank-sum test was used to compare the difference as normality was not satisfied, * $P<0.05$)

Table S3-20: Univariate analysis of dynamic network characteristic

Item	P_{50}_PC	IQR_PC	P_{50}_PE	IQR_PE	W	P
Mean-Little world index	1.01	0.05	1.06	0.07	56.0	0.001*
Standard Deviation-Little world index	0.12	0.03	0.13	0.04	129.0	0.336
P_{50}-Little world index	1.00	0.05	1.05	0.07	65.0	0.002*
IQR-Little world index	0.15	0.05	0.16	0.03	124.0	0.262
Mean-Average vertex strength	5.52	0.21	5.96	0.31	17.0	<0.001*
Standard Deviation-Average vertex strength	0.29	0.10	0.30	0.11	150.0	0.765
P_{50}-Average vertex strength	5.46	0.23	5.97	0.35	19.0	<0.001*
IQR-Average vertex strength	0.36	0.14	0.37	0.18	137.0	0.479
Mean-Average path length	1.86	0.03	1.86	0.05	134.0	0.422
Standard Deviation-Average path length	0.11	0.05	0.12	0.06	109.0	0.109
P_{50}-Average path length	1.85	0.03	1.86	0.04	113.5	0.143
IQR-Average path length	0.11	0.06	0.14	0.06	105.0	0.082
Mean-Transitivity	0.56	0.04	0.61	0.06	77.0	0.007*
Standard Deviation-Transitivity	0.07	0.02	0.07	0.01	122.0	0.236
P_{50}-Transitivity	0.56	0.04	0.61	0.06	84.0	0.015*
IQR-Transitivity	0.09	0.02	0.09	0.02	123.0	0.249
Mean-Diameter	2.12	0.27	2.51	0.34	45.0	<0.001*
Standard Deviation-Diameter	0.43	0.09	0.52	0.17	93.0	0.033*
P_{50}-Diameter	2.07	0.24	2.42	0.34	43.0	<0.001*
IQR-Diameter	0.55	0.15	0.63	0.21	120.0	0.211

(CORR as the connectivity method in Beta frequency band, the rank-sum test was used to compare the difference as normality was not satisfied, * P<0.05)

Table S3-21: Univariate analysis of dynamic network characteristic

Item	P_{50}_PC	*IQR*_PC	P_{50}_PE	*IQR*_PE	*W*	*P*
Mean-Little world index	1.14	0.04	1.17	0.05	71.0	0.004*
Standard Deviation-Little world index	0.17	0.02	0.19	0.02	64.0	0.002*
P_{50}-Little world index	1.13	0.05	1.16	0.03	60.0	0.001*
IQR-Little world index	0.23	0.04	0.26	0.02	82.0	0.012*
Mean-Average vertex strength	1.75	0.49	2.28	0.61	56.0	0.001*
Standard Deviation-Average vertex strength	0.29	0.13	0.47	0.23	52.0	<0.001*
P_{50}-Average vertex strength	1.66	0.48	2.24	0.53	55.5	0.001*
IQR-Average vertex strength	0.37	0.08	0.60	0.32	40.0	<0.001*
Mean-Average path length	1.64	0.01	1.64	0.01	122.0	0.236
Standard Deviation-Average path length	0.04	0.01	0.04	0.01	81.0	0.011*
P_{50}-Average path length	1.63	0.01	1.64	0.01	103.0	0.061
IQR-Average path length	0.04	0.01	0.05	0.01	84.5	0.016*
Mean-Transitivity	0.44	0.01	0.45	0.02	65.0	0.002*
Standard Deviation-Transitivity	0.05	0.01	0.05	0.01	67.0	0.002*
P_{50}-Transitivity	0.44	0.01	0.45	0.02	60.5	0.002*
IQR-Transitivity	0.06	0.01	0.07	0.01	59.0	0.001*
Mean-Diameter	0.64	0.17	0.85	0.24	54.0	<0.001*
Standard Deviation-Diameter	0.14	0.07	0.23	0.10	52.0	<0.001*
P_{50}-Diameter	0.61	0.17	0.84	0.25	50.5	0.001*
IQR-Diameter	0.18	0.07	0.26	0.13	53.5	0.001*

(PLI as the connectivity method in full frequency band, the rank-sum test was used to compare the difference as normality was not satisfied, * $P<0.05$)

Table S3-22: Univariate analysis of dynamic network characteristic

Item	P_{50}_PC	IQR_PC	P_{50}_PE	IQR_PE	W	P
Mean-Little world index	0.93	0.07	1.00	0.08	26.0	<0.001*
Standard Deviation-Little world index	0.10	0.02	0.13	0.04	53.0	<0.001*
P_{50}-Little world index	0.92	0.07	1.00	0.09	30.0	<0.001*
IQR-Little world index	0.13	0.03	0.16	0.04	74.0	0.005*
Mean-Average vertex strength	4.16	0.20	4.61	0.42	16.0	<0.001*
Standard Deviation-Average vertex strength	0.12	0.05	0.27	0.15	1.0	<0.001*
P_{50}-Average vertex strength	4.14	0.19	4.58	0.41	18.0	<0.001*
IQR-Average vertex strength	0.14	0.07	0.32	0.18	17.0	<0.001*
Mean-Average path length	1.82	0.04	1.82	0.01	130.0	0.352
Standard Deviation-Average path length	0.06	0.03	0.08	0.04	64.0	0.002*
P_{50}-Average path length	1.81	0.04	1.82	0.02	122.0	0.230
IQR-Average path length	0.07	0.03	0.10	0.04	74.0	0.006*
Mean-Transitivity	0.48	0.06	0.53	0.04	56.0	0.001*
Standard Deviation-Transitivity	0.05	0.01	0.06	0.01	69.0	0.003*
P_{50}-Transitivity	0.48	0.05	0.53	0.04	64.0	0.002*
IQR-Transitivity	0.07	0.02	0.07	0.02	138.0	0.498
Mean-Diameter	1.16	0.18	1.54	0.32	23.0	<0.001*
Standard Deviation-Diameter	0.18	0.05	0.32	0.17	25.0	<0.001*
P_{50}-Diameter	1.13	0.19	1.46	0.27	25.0	<0.001*
IQR-Diameter	0.23	0.08	0.35	0.17	37.0	<0.001*

(MSC as the connectivity method in full frequency band, the rank-sum test was used to compare the difference as normality was not satisfied, * P<0.05)

Table S3-23: Univariate analysis of dynamic network characteristic

Item	P_{50}_PC	*IQR*_PC	P_{50}_PE	*IQR*_PE	*W*	*P*
Mean-Little world index	1.08	0.02	1.09	0.05	96.0	0.042*
Standard Deviation-Little world index	0.10	0.01	0.13	0.02	31.0	<0.001*
P_{50}-Little world index	1.07	0.03	1.09	0.04	77.0	0.007*
IQR-Little world index	0.14	0.04	0.17	0.03	51.0	<0.001*
Mean-Average vertex strength	0.72	0.02	1.08	0.26	0.0	<0.001*
Standard Deviation-Average vertex strength	0.10	0.02	0.25	0.17	0.0	<0.001*
P_{50}-Average vertex strength	0.71	0.03	1.03	0.23	0.0	<0.001*
IQR-Average vertex strength	0.12	0.03	0.35	0.18	0.0	<0.001*
Mean-Average path length	1.57	0.00	1.58	0.01	71.0	0.005*
Standard Deviation-Average path length	0.02	0.00	0.03	0.01	38.0	<0.001*
P_{50}-Average path length	1.57	0.01	1.57	0.01	101.5	0.049*
IQR-Average path length	0.02	0.01	0.03	0.01	38.0	<0.001*
Mean-Transitivity	0.46	0.01	0.46	0.01	98.0	0.049*
Standard Deviation-Transitivity	0.04	0.00	0.04	0.01	39.0	<0.001*
P_{50}-Transitivity	0.46	0.01	0.47	0.01	70.5	0.005*
IQR-Transitivity	0.05	0.01	0.05	0.01	64.0	0.002*
Mean-Diameter	0.22	0.01	0.32	0.09	0.0	<0.001*
Standard Deviation-Diameter	0.04	0.01	0.09	0.06	0.0	<0.001*
P_{50}-Diameter	0.21	0.01	0.31	0.07	0.0	<0.001*
IQR-Diameter	0.04	0.00	0.11	0.04	0.0	<0.001*

(iCOH as the connectivity method in full frequency band, the rank-sum test was used to compare the difference as normality was not satisfied, * $P<0.05$)

Table S3-24: Univariate analysis of dynamic network characteristic

Item	P_{50}_PC	*IQR*_PC	P_{50}_PE	*IQR*_PE	*W*	*P*
Mean-Little world index	1.01	0.05	1.06	0.07	61.0	0.001*
Standard Deviation-Little world index	0.12	0.04	0.13	0.04	129.0	0.336
P_{50}-Little world index	1.00	0.04	1.05	0.07	62.0	0.001*
IQR-Little world index	0.15	0.05	0.16	0.04	123.0	0.249
Mean-Average vertex strength	5.51	0.21	5.95	0.31	17.0	<0.001*
Standard Deviation-Average vertex strength	0.29	0.10	0.30	0.11	149.0	0.741
P_{50}-Average vertex strength	5.45	0.23	5.97	0.35	19.0	<0.001*
IQR-Average vertex strength	0.35	0.14	0.37	0.18	137.0	0.479
Mean-Average path length	1.86	0.04	1.86	0.05	135.0	0.440
Standard Deviation-Average path length	0.11	0.05	0.13	0.06	108.0	0.102
P_{50}-Average path length	1.85	0.03	1.86	0.03	114.5	0.151
IQR-Average path length	0.11	0.06	0.14	0.06	106.5	0.091
Mean-Transitivity	0.56	0.04	0.61	0.06	78.0	0.008*
Standard Deviation-Transitivity	0.07	0.02	0.07	0.01	122.0	0.236
P_{50}-Transitivity	0.56	0.04	0.61	0.06	84.0	0.015*
IQR-Transitivity	0.09	0.02	0.09	0.02	119.0	0.200
Mean-Diameter	2.12	0.26	2.51	0.34	43.0	<0.001*
Standard Deviation-Diameter	0.43	0.10	0.53	0.18	91.0	0.028*
P_{50}-Diameter	2.07	0.21	2.42	0.33	42.0	<0.001*
IQR-Diameter	0.59	0.14	0.63	0.14	116.0	0.168

(CORR as the connectivity method in full frequency band, the rank-sum test was used to compare the difference as normality was not satisfied, * $P<0.05$)

Supplementary Part S4

Univariate analysis of dynamic network characteristics-Split segment EEG signals dataset

Table S4-1: Univariate analysis of dynamic network characteristic

Item	P_{50}_PC	*IQR*_PC	P_{50}_PE	*IQR*_PE	*W*	*P*
Mean-Little world index	1.14	0.03	1.15	0.05	1658.0	0.015*
Standard Deviation-Little world index	0.17	0.02	0.18	0.03	1421.0	<0.001*
P_{50}-Little world index	1.13	0.05	1.14	0.05	1642.0	0.012*
IQR-Little world index	0.23	0.04	0.24	0.05	1638.0	0.012*
Mean-Average vertex strength	2.60	0.22	2.84	0.39	1008.0	<0.001*
Standard Deviation-Average vertex strength	0.39	0.11	0.49	0.18	897.0	<0.001*
P_{50}-Average vertex strength	2.55	0.23	2.80	0.29	1030.5	<0.001*
IQR-Average vertex strength	0.52	0.11	0.65	0.30	843.5	<0.001*
Mean-Average path length	1.64	0.01	1.64	0.01	1751.0	0.045*
Standard Deviation-Average path length	0.04	0.01	0.04	0.01	1143.0	<0.001*
P_{50}-Average path length	1.63	0.01	1.64	0.01	1503.5	0.001*
IQR-Average path length	0.04	0.01	0.05	0.01	1540.0	0.003*
Mean-Transitivity	0.44	0.01	0.45	0.01	1072.0	<0.001*
Standard Deviation-Transitivity	0.05	0.01	0.05	0.01	1354.0	<0.001*
P_{50}-Transitivity	0.44	0.01	0.45	0.01	1100.0	<0.001*
IQR-Transitivity	0.06	0.01	0.06	0.01	1576.0	0.005*
Mean-Diameter	0.96	0.09	1.06	0.17	947.0	<0.001*
Standard Deviation-Diameter	0.20	0.05	0.24	0.09	934.0	<0.001*
P_{50}-Diameter	0.93	0.09	1.03	0.14	903.5	<0.001*
IQR-Diameter	0.25	0.07	0.31	0.11	898.0	<0.001*

(PLI as the connectivity method in Delta frequency band, the rank-sum test was used to compare the difference as normality was not satisfied, * $P<0.05$)

Table S4-2: Univariate analysis of dynamic network characteristic

Item	P_{50}_PC	*IQR*_PC	P_{50}_PE	*IQR*_PE	*W*	*P*
Mean-Little world index	1.06	0.03	1.09	0.04	1165.0	<0.001*
Standard Deviation-Little world index	0.13	0.02	0.14	0.03	1725.0	0.034*
P_{50}-Little world index	1.05	0.03	1.08	0.04	1105.0	<0.001*
IQR-Little world index	0.17	0.03	0.18	0.03	1722.0	0.033*
Mean-Average vertex strength	5.16	0.21	5.50	0.30	349.0	<0.001*
Standard Deviation-Average vertex strength	0.41	0.08	0.43	0.21	1540.0	0.003*
P_{50}-Average vertex strength	5.10	0.19	5.46	0.34	454.0	<0.001*
IQR-Average vertex strength	0.54	0.14	0.61	0.26	1499.0	0.002*
Mean-Average path length	1.87	0.03	1.87	0.04	2129.0	0.764
Standard Deviation-Average path length	0.14	0.04	0.15	0.04	1464.0	0.001*
P_{50}-Average path length	1.87	0.02	1.86	0.04	1950.5	0.267
IQR-Average path length	0.13	0.03	0.15	0.05	1374.0	<0.001*
Mean-Transitivity	0.58	0.03	0.61	0.04	1042.0	<0.001*
Standard Deviation-Transitivity	0.07	0.01	0.08	0.01	1979.0	0.328
P_{50}-Transitivity	0.58	0.03	0.60	0.04	1140.0	<0.001*
IQR-Transitivity	0.10	0.02	0.10	0.03	1936.0	0.241
Mean-Diameter	1.87	0.15	2.12	0.25	410.0	<0.001*
Standard Deviation-Diameter	0.44	0.10	0.53	0.15	1102.0	<0.001*
P_{50}-Diameter	1.80	0.13	2.04	0.25	495.0	<0.001*
IQR-Diameter	0.58	0.12	0.65	0.22	1418.0	<0.001*

(MSC as the connectivity method in Delta frequency band, the rank-sum test was used to compare the difference as normality was not satisfied, * $P<0.05$)

Table S4-3: Univariate analysis of dynamic network characteristic

Item	P_{50}_PC	IQR_PC	P_{50}_PE	IQR_PE	W	P
Mean-Little world index	1.05	0.03	1.04	0.06	2537.0	0.124
Standard Deviation-Little world index	0.14	0.02	0.16	0.03	695.0	<0.001*
P_{50}-Little world index	1.05	0.03	1.04	0.05	2518.0	0.147
IQR-Little world index	0.17	0.03	0.22	0.04	712.0	<0.001*
Mean-Average vertex strength	2.01	0.13	2.14	0.31	1251.0	<0.001*
Standard Deviation-Average vertex strength	0.33	0.07	0.42	0.15	680.0	<0.001*
P_{50}-Average vertex strength	1.96	0.12	2.07	0.28	1235.0	<0.001*
IQR-Average vertex strength	0.43	0.10	0.57	0.25	813.0	<0.001*
Mean-Average path length	1.58	0.00	1.58	0.00	1648.5	0.014*
Standard Deviation-Average path length	0.02	0.01	0.03	0.01	1056.0	<0.001*
P_{50}-Average path length	1.57	0.00	1.57	0.01	1869.0	0.096
IQR-Average path length	0.02	0.01	0.03	0.01	1130.5	<0.001*
Mean-Transitivity	0.45	0.01	0.45	0.01	2797.0	0.007*
Standard Deviation-Transitivity	0.04	0.01	0.05	0.01	826.0	<0.001*
P_{50}-Transitivity	0.45	0.01	0.45	0.01	2444.0	0.264
IQR-Transitivity	0.06	0.01	0.07	0.02	1141.0	<0.001*
Mean-Diameter	0.63	0.04	0.67	0.10	1164.0	<0.001*
Standard Deviation-Diameter	0.13	0.03	0.16	0.06	690.0	<0.001*
P_{50}-Diameter	0.61	0.05	0.64	0.09	1274.0	<0.001*
IQR-Diameter	0.16	0.05	0.21	0.08	688.0	<0.001*

(iCOH as the connectivity method in Delta frequency band, the rank-sum test was used to compare the difference as normality was not satisfied, * $P<0.05$)

Table S4-4: Univariate analysis of dynamic network characteristic

Item	P_{50}_PC	IQR_PC	P_{50}_PE	IQR_PE	W	P
Mean-Little world index	1.03	0.05	1.06	0.06	1461.0	0.001*
Standard Deviation-Little world index	0.13	0.03	0.12	0.02	2372.0	0.428
P_{50}-Little world index	1.03	0.04	1.05	0.06	1305.0	<0.001*
IQR-Little world index	0.17	0.04	0.16	0.04	2309.0	0.611
Mean-Average vertex strength	5.76	0.21	6.03	0.29	694.0	<0.001*
Standard Deviation-Average vertex strength	0.33	0.09	0.33	0.14	2009.0	0.400
P_{50}-Average vertex strength	5.74	0.21	6.01	0.32	736.0	<0.001*
IQR-Average vertex strength	0.42	0.13	0.45	0.18	2105.0	0.683
Mean-Average path length	1.86	0.04	1.87	0.04	1608.0	0.008*
Standard Deviation-Average path length	0.13	0.05	0.14	0.05	1977.0	0.324
P_{50}-Average path length	1.85	0.03	1.87	0.03	1569.0	0.005*
IQR-Average path length	0.13	0.04	0.13	0.04	2039.0	0.480
Mean-Transitivity	0.58	0.04	0.60	0.05	1733.0	0.037*
Standard Deviation-Transitivity	0.07	0.02	0.08	0.02	2169.0	0.905
P_{50}-Transitivity	0.58	0.04	0.59	0.05	1806.5	0.079
IQR-Transitivity	0.10	0.03	0.10	0.03	2286.0	0.686
Mean-Diameter	2.33	0.19	2.49	0.31	1044.0	<0.001*
Standard Deviation-Diameter	0.48	0.11	0.53	0.11	1527.0	0.003*
P_{50}-Diameter	2.28	0.18	2.42	0.26	1058.0	<0.001*
IQR-Diameter	0.59	0.16	0.63	0.16	1753.0	0.046*

(CORR as the connectivity method in Delta frequency band, the rank-sum test was used to compare the difference as normality was not satisfied, * P<0.05)

Table S4-5: Univariate analysis of dynamic network characteristic

Item	P_{50}_PC	IQR_PC	P_{50}_PE	IQR_PE	W	P
Mean-Little world index	1.14	0.03	1.15	0.05	1679.0	0.020*
Standard Deviation-Little world index	0.17	0.02	0.18	0.02	1518.0	0.002*
P_{50}-Little world index	1.13	0.03	1.14	0.06	1628.0	0.010*
IQR-Little world index	0.23	0.05	0.24	0.04	1769.0	0.054
Mean-Average vertex strength	2.17	0.40	2.54	0.54	889.0	<0.001*
Standard Deviation-Average vertex strength	0.34	0.12	0.46	0.18	861.0	<0.001*
P_{50}-Average vertex strength	2.10	0.36	2.45	0.47	848.0	<0.001*
IQR-Average vertex strength	0.42	0.17	0.61	0.24	827.5	<0.001*
Mean-Average path length	1.64	0.01	1.64	0.01	1912.0	0.200
Standard Deviation-Average path length	0.04	0.01	0.04	0.01	1323.0	<0.001*
P_{50}-Average path length	1.64	0.01	1.64	0.01	1805.5	0.066
IQR-Average path length	0.04	0.01	0.05	0.01	1350.5	<0.001*
Mean-Transitivity	0.44	0.01	0.45	0.01	1217.0	<0.001*
Standard Deviation-Transitivity	0.05	0.01	0.05	0.01	1453.0	0.001 *
P_{50}-Transitivity	0.44	0.01	0.45	0.01	1307.0	<0.001*
IQR-Transitivity	0.06	0.01	0.06	0.02	1796.0	0.071
Mean-Diameter	0.79	0.16	0.93	0.21	889.0	<0.001*
Standard Deviation-Diameter	0.17	0.06	0.23	0.10	928.0	<0.001*
P_{50}-Diameter	0.76	0.13	0.89	0.19	900.5	<0.001*
IQR-Diameter	0.20	0.06	0.29	0.13	807.5	<0.001*

(PLI as the connectivity method in Theta frequency band, the rank-sum test was used to compare the difference as normality was not satisfied, * $P<0.05$)

Table S4-6: Univariate analysis of dynamic network characteristic

Item	P_{50}_PC	*IQR*_PC	P_{50}_PE	*IQR*_PE	*W*	*P*
Mean-Little world index	1.04	0.04	1.08	0.06	835.0	<0.001*
Standard Deviation-Little world index	0.12	0.03	0.14	0.03	1316.0	<0.001*
P_{50}-Little world index	1.03	0.04	1.08	0.06	922.0	<0.001*
IQR-Little world index	0.16	0.04	0.17	0.04	1737.0	0.038*
Mean-Average vertex strength	4.97	0.30	5.51	0.50	487.0	<0.001*
Standard Deviation-Average vertex strength	0.36	0.11	0.44	0.14	950.0	<0.001*
P_{50}-Average vertex strength	4.92	0.27	5.50	0.54	497.0	<0.001*
IQR-Average vertex strength	0.45	0.14	0.59	0.19	826.0	<0.001*
Mean-Average path length	1.86	0.03	1.87	0.05	1903.0	0.187
Standard Deviation-Average path length	0.11	0.04	0.15	0.06	1001.0	<0.001*
P_{50}-Average path length	1.85	0.02	1.86	0.06	1602.5	0.007*
IQR-Average path length	0.12	0.04	0.15	0.06	979.5	<0.001*
Mean-Transitivity	0.56	0.04	0.62	0.05	658.0	<0.001*
Standard Deviation-Transitivity	0.07	0.01	0.08	0.01	1537.0	0.003*
P_{50}-Transitivity	0.56	0.04	0.61	0.05	661.0	<0.001*
IQR-Transitivity	0.09	0.02	0.10	0.02	1462.0	0.001*
Mean-Diameter	1.74	0.24	2.20	0.36	492.0	<0.001*
Standard Deviation-Diameter	0.39	0.11	0.52	0.10	741.0	<0.001*
P_{50}-Diameter	1.68	0.22	2.09	0.36	499.0	<0.001*
IQR-Diameter	0.49	0.17	0.63	0.17	882.0	<0.001*

(MSC as the connectivity method in Theta frequency band, the rank-sum test was used to compare the difference as normality was not satisfied, * $P<0.05$)

Table S4-7: Univariate analysis of dynamic network characteristic

Item	P_{50}_PC	*IQR*_PC	P_{50}_PE	*IQR*_PE	*W*	*P*
Mean-Little world index	1.07	0.02	1.06	0.04	2358.0	0.466
Standard Deviation-Little world index	0.13	0.02	0.15	0.04	974.0	<0.001*
P_{50}-Little world index	1.06	0.02	1.06	0.05	2343.0	0.508
IQR-Little world index	0.16	0.03	0.19	0.05	1064.0	<0.001*
Mean-Average vertex strength	2.02	0.17	2.33	0.37	743.0	<0.001*
Standard Deviation-Average vertex strength	0.34	0.09	0.48	0.15	565.0	<0.001*
P_{50}-Average vertex strength	1.98	0.17	2.26	0.42	731.0	<0.001*
IQR-Average vertex strength	0.45	0.10	0.63	0.21	678.0	<0.001*
Mean-Average path length	1.58	0.00	1.58	0.01	1418.0	<0.001*
Standard Deviation-Average path length	0.02	0.01	0.03	0.01	664.0	<0.001*
P_{50}-Average path length	1.57	0.00	1.58	0.01	1472.5	<0.001*
IQR-Average path length	0.02	0.01	0.04	0.01	877.0	<0.001*
Mean-Transitivity	0.46	0.01	0.46	0.01	2253.0	0.799
Standard Deviation-Transitivity	0.04	0.01	0.05	0.01	969.0	<0.001*
P_{50}-Transitivity	0.46	0.01	0.46	0.01	1852.5	0.121
IQR-Transitivity	0.05	0.01	0.06	0.02	1626.0	0.010*
Mean-Diameter	0.63	0.06	0.73	0.12	710.0	<0.001*
Standard Deviation-Diameter	0.13	0.03	0.18	0.05	555.0	<0.001*
P_{50}-Diameter	0.61	0.05	0.70	0.14	796.0	<0.001*
IQR-Diameter	0.16	0.04	0.22	0.08	640.0	<0.001*

(iCOH as the connectivity method in Theta frequency band, the rank-sum test was used to compare the difference as normality was not satisfied, * $P<0.05$)

Table S4-8: Univariate analysis of dynamic network characteristic

Item	P_{50}_PC	*IQR*_PC	P_{50}_PE	*IQR*_PE	*W*	*P*
Mean-Little world index	1.02	0.05	1.05	0.05	1268.0	<0.001*
Standard Deviation-Little world index	0.12	0.04	0.12	0.03	2330.0	0.547
P_{50}-Little world index	1.01	0.07	1.05	0.05	1247.0	<0.001*
IQR-Little world index	0.16	0.05	0.16	0.03	2123.0	0.743
Mean-Average vertex strength	5.62	0.19	5.95	0.30	460.0	<0.001*
Standard Deviation-Average vertex strength	0.30	0.09	0.30	0.14	1948.0	0.264
P_{50}-Average vertex strength	5.60	0.16	5.93	0.32	498.0	<0.001*
IQR-Average vertex strength	0.39	0.13	0.38	0.20	2027.0	0.447
Mean-Average path length	1.86	0.05	1.87	0.05	1685.0	0.021*
Standard Deviation-Average path length	0.12	0.06	0.12	0.04	2200.0	0.987
P_{50}-Average path length	1.85	0.03	1.87	0.04	1587.0	0.006*
IQR-Average path length	0.12	0.04	0.12	0.04	2158.5	0.867
Mean-Transitivity	0.57	0.05	0.59	0.05	1694.0	0.024*
Standard Deviation-Transitivity	0.07	0.02	0.07	0.02	2094.0	0.647
P_{50}-Transitivity	0.57	0.05	0.58	0.05	1762.0	0.050
IQR-Transitivity	0.10	0.02	0.10	0.04	2306.0	0.621
Mean-Diameter	2.21	0.21	2.41	0.34	905.0	<0.001*
Standard Deviation-Diameter	0.44	0.12	0.50	0.16	1563.0	0.004*
P_{50}-Diameter	2.15	0.20	2.37	0.31	839.0	<0.001*
IQR-Diameter	0.55	0.12	0.62	0.23	1530.0	0.003*

(CORR as the connectivity method in Theta frequency band, the rank-sum test was used to compare the difference as normality was not satisfied, * $P<0.05$)

Table S4-9: Univariate analysis of dynamic network characteristic

Item	P_{50}_PC	IQR_PC	P_{50}_PE	IQR_PE	W	P
Mean-Little world index	1.14	0.03	1.15	0.04	1651.0	0.014*
Standard Deviation-Little world index	0.18	0.02	0.19	0.02	1502.0	0.002*
P_{50}-Little world index	1.13	0.04	1.14	0.05	1527.0	0.003*
IQR-Little world index	0.23	0.04	0.25	0.04	1555.0	0.004*
Mean-Average vertex strength	2.00	0.42	2.48	0.58	821.0	<0.001*
Standard Deviation-Average vertex strength	0.34	0.11	0.47	0.18	815.0	<0.001*
P_{50}-Average vertex strength	1.94	0.40	2.39	0.52	815.5	<0.001*
IQR-Average vertex strength	0.42	0.14	0.61	0.26	826.5	<0.001*
Mean-Average path length	1.64	0.01	1.64	0.01	1584.0	0.006*
Standard Deviation-Average path length	0.04	0.01	0.04	0.01	1363.0	<0.001*
P_{50}-Average path length	1.63	0.01	1.64	0.01	1440.5	<0.001*
IQR-Average path length	0.04	0.01	0.05	0.01	1719.0	0.030*
Mean-Transitivity	0.44	0.01	0.45	0.01	1256.0	<0.001*
Standard Deviation-Transitivity	0.05	0.01	0.05	0.01	1555.0	0.004*
P_{50}-Transitivity	0.44	0.01	0.45	0.01	1404.5	<0.001*
IQR-Transitivity	0.06	0.01	0.06	0.02	1515.0	0.002*
Mean-Diameter	0.73	0.16	0.92	0.22	764.0	<0.001*
Standard Deviation-Diameter	0.16	0.05	0.22	0.12	825.0	<0.001*
P_{50}-Diameter	0.70	0.15	0.88	0.21	753.5	<0.001*
IQR-Diameter	0.20	0.06	0.28	0.15	890.5	<0.001*

(PLI as the connectivity method in Alpha-1 frequency band, the rank-sum test was used to compare the difference as normality was not satisfied, * $P<0.05$)

Table S4-10: Univariate analysis of dynamic network characteristic

Item	P_{50}_PC	*IQR*_PC	P_{50}_PE	*IQR*_PE	*W*	*P*
Mean-Little world index	1.06	0.05	1.11	0.07	1045.0	<0.001*
Standard Deviation-Little world index	0.13	0.02	0.16	0.04	936.0	<0.001*
P_{50}-Little world index	1.05	0.04	1.09	0.07	1202.0	<0.001*
IQR-Little world index	0.16	0.03	0.19	0.05	1346.0	<0.001*
Mean-Average vertex strength	5.19	0.60	5.71	0.46	981.0	<0.001*
Standard Deviation-Average vertex strength	0.42	0.14	0.53	0.19	858.0	<0.001*
P_{50}-Average vertex strength	5.10	0.61	5.68	0.54	1031.0	<0.001*
IQR-Average vertex strength	0.54	0.23	0.72	0.28	1164.0	<0.001*
Mean-Average path length	1.87	0.03	1.85	0.05	2885.0	0.002*
Standard Deviation-Average path length	0.12	0.05	0.17	0.05	997.0	<0.001*
P_{50}-Average path length	1.86	0.03	1.86	0.04	2315.5	0.591
IQR-Average path length	0.12	0.05	0.18	0.05	1060.5	<0.001*
Mean-Transitivity	0.57	0.06	0.62	0.05	1087.0	<0.001*
Standard Deviation-Transitivity	0.07	0.01	0.08	0.02	1223.0	<0.001*
P_{50}-Transitivity	0.57	0.06	0.63	0.06	1122.5	<0.001*
IQR-Transitivity	0.09	0.02	0.10	0.02	1347.0	<0.001*
Mean-Diameter	1.88	0.45	2.27	0.32	1068.0	<0.001*
Standard Deviation-Diameter	0.47	0.18	0.57	0.10	1112.0	<0.001*
P_{50}-Diameter	1.79	0.48	2.16	0.35	1102.0	<0.001*
IQR-Diameter	0.59	0.26	0.69	0.19	1429.0	0.001*

(MSC as the connectivity method in Alpha-1 frequency band, the rank-sum test was used to compare the difference as normality was not satisfied, * $P<0.05$)

Table S4-11: Univariate analysis of dynamic network characteristic

Item	P_{50}_PC	*IQR*_PC	P_{50}_PE	*IQR*_PE	*W*	*P*
Mean-Little world index	1.05	0.03	1.04	0.05	2863.0	0.003*
Standard Deviation-Little world index	0.14	0.03	0.16	0.03	777.0	<0.001*
P_{50}-Little world index	1.05	0.03	1.03	0.05	2730.0	0.016*
IQR-Little world index	0.17	0.03	0.21	0.06	1217.0	<0.001*
Mean-Average vertex strength	2.48	0.10	2.85	0.58	710.0	<0.001*
Standard Deviation-Average vertex strength	0.41	0.10	0.59	0.24	516.0	<0.001*
P_{50}-Average vertex strength	2.43	0.10	2.76	0.50	733.0	<0.001*
IQR-Average vertex strength	0.55	0.17	0.82	0.30	690.0	<0.001*
Mean-Average path length	1.58	0.00	1.58	0.00	1820.0	0.090
Standard Deviation-Average path length	0.03	0.01	0.03	0.01	856.0	<0.001*
P_{50}-Average path length	1.57	0.01	1.57	0.01	1618.5	0.004*
IQR-Average path length	0.02	0.01	0.04	0.01	1085.0	<0.001*
Mean-Transitivity	0.45	0.01	0.45	0.01	3079.0	<0.001*
Standard Deviation-Transitivity	0.04	0.01	0.05	0.01	958.0	<0.001*
P_{50}-Transitivity	0.45	0.01	0.45	0.01	2858.5	0.003*
IQR-Transitivity	0.06	0.01	0.07	0.02	1282.0	<0.001*
Mean-Diameter	0.77	0.04	0.89	0.20	727.0	<0.001*
Standard Deviation-Diameter	0.16	0.04	0.23	0.08	517.0	<0.001*
P_{50}-Diameter	0.74	0.04	0.85	0.18	823.0	<0.001*
IQR-Diameter	0.20	0.06	0.30	0.11	678.0	<0.001*

(iCOH as the connectivity method in Alpha-1 frequency band, the rank-sum test was used to compare the difference as normality was not satisfied, * $P<0.05$)

Table S4-12: Univariate analysis of dynamic network characteristic

Item	P_{50}_PC	*IQR*_PC	P_{50}_PE	*IQR*_PE	*W*	*P*
Mean-Little world index	1.02	0.05	1.05	0.05	1314.0	<0.001*
Standard Deviation-Little world index	0.12	0.03	0.12	0.03	2163.0	0.883
P_{50}-Little world index	1.01	0.05	1.04	0.05	1321.0	<0.001*
IQR-Little world index	0.16	0.05	0.16	0.03	2164.0	0.887
Mean-Average vertex strength	5.61	0.19	5.94	0.31	450.0	<0.001*
Standard Deviation-Average vertex strength	0.29	0.08	0.30	0.14	1844.0	0.112
P_{50}-Average vertex strength	5.58	0.19	5.92	0.32	488.0	<0.001*
IQR-Average vertex strength	0.38	0.12	0.38	0.22	2000.0	0.377
Mean-Average path length	1.87	0.05	1.87	0.04	1787.0	0.065
Standard Deviation-Average path length	0.11	0.05	0.11	0.04	2073.0	0.580
P_{50}-Average path length	1.85	0.04	1.86	0.04	1766.5	0.052
IQR-Average path length	0.13	0.05	0.12	0.04	2234.0	0.865
Mean-Transitivity	0.57	0.05	0.59	0.05	1674.0	0.019*
Standard Deviation-Transitivity	0.07	0.02	0.07	0.02	1921.0	0.215
P_{50}-Transitivity	0.57	0.05	0.58	0.05	1722.5	0.033*
IQR-Transitivity	0.09	0.02	0.09	0.04	2115.0	0.716
Mean-Diameter	2.20	0.20	2.40	0.32	913.0	<0.001*
Standard Deviation-Diameter	0.43	0.12	0.50	0.17	1472.0	0.001*
P_{50}-Diameter	2.16	0.23	2.35	0.31	946.0	<0.001*
IQR-Diameter	0.54	0.15	0.61	0.20	1617.0	0.009*

(CORR as the connectivity method in Alpha-1 frequency band, the rank-sum test was used to compare the difference as normality was not satisfied, * $P<0.05$)

Table S4-13: Univariate analysis of dynamic network characteristic

Item	P_{50}_PC	IQR_PC	P_{50}_PE	IQR_PE	W	P
Mean-Little world index	1.14	0.04	1.15	0.06	1751.0	0.045*
Standard Deviation-Little world index	0.17	0.03	0.19	0.02	1412.0	<0.001*
P_{50}-Little world index	1.14	0.05	1.13	0.06	1976.0	0.322
IQR-Little world index	0.23	0.04	0.24	0.04	1698.0	0.025*
Mean-Average vertex strength	1.92	0.41	2.46	0.54	782.0	<0.001*
Standard Deviation-Average vertex strength	0.33	0.11	0.46	0.19	809.0	<0.001*
P_{50}-Average vertex strength	1.86	0.40	2.34	0.47	785.5	<0.001*
IQR-Average vertex strength	0.40	0.13	0.63	0.30	819.5	<0.001*
Mean-Average path length	1.64	0.01	1.64	0.01	1659.0	0.015*
Standard Deviation-Average path length	0.04	0.01	0.04	0.02	1607.0	0.008*
P_{50}-Average path length	1.64	0.01	1.64	0.01	1504.5	0.001*
IQR-Average path length	0.05	0.01	0.05	0.01	2012.5	0.402
Mean-Transitivity	0.44	0.01	0.45	0.01	1251.0	<0.001*
Standard Deviation-Transitivity	0.05	0.01	0.05	0.01	1449.0	0.001*
P_{50}-Transitivity	0.44	0.01	0.45	0.01	1317.5	<0.001*
IQR-Transitivity	0.06	0.01	0.06	0.01	1612.0	0.008*
Mean-Diameter	0.71	0.16	0.90	0.23	767.5	<0.001*
Standard Deviation-Diameter	0.16	0.05	0.22	0.11	815.0	<0.001*
P_{50}-Diameter	0.68	0.16	0.87	0.20	736.0	<0.001*
IQR-Diameter	0.19	0.07	0.28	0.11	781.0	<0.001*

(PLI as the connectivity method in Alpha-2 frequency band, the rank-sum test was used to compare the difference as normality was not satisfied, * P<0.05)

Table S4-14: Univariate analysis of dynamic network characteristic

Item	P_{50}_PC	*IQR*_PC	P_{50}_PE	*IQR*_PE	*W*	*P*
Mean-Little world index	1.05	0.08	1.11	0.06	1507.0	0.002*
Standard Deviation-Little world index	0.13	0.04	0.15	0.04	1487.0	0.001*
P_{50}-Little world index	1.05	0.07	1.09	0.06	1513.0	0.002*
IQR-Little world index	0.17	0.03	0.19	0.06	1510.0	0.002*
Mean-Average vertex strength	5.15	0.76	5.68	0.41	1466.0	0.001*
Standard Deviation-Average vertex strength	0.44	0.17	0.55	0.26	1153.0	<0.001*
P_{50}-Average vertex strength	5.10	0.83	5.63	0.47	1560.0	0.004*
IQR-Average vertex strength	0.58	0.24	0.70	0.40	1461.0	0.001*
Mean-Average path length	1.86	0.02	1.86	0.06	2362.0	0.455
Standard Deviation-Average path length	0.13	0.07	0.17	0.05	1447.0	0.001*
P_{50}-Average path length	1.85	0.03	1.86	0.05	2112.5	0.707
IQR-Average path length	0.13	0.08	0.17	0.06	1509.5	0.002*
Mean-Transitivity	0.58	0.08	0.62	0.05	1553.0	0.004*
Standard Deviation-Transitivity	0.08	0.01	0.08	0.02	1435.0	0.001*
P_{50}-Transitivity	0.57	0.08	0.61	0.06	1581.0	0.006*
IQR-Transitivity	0.10	0.03	0.10	0.03	1762.0	0.050
Mean-Diameter	1.89	0.58	2.26	0.26	1490.0	0.001*
Standard Deviation-Diameter	0.49	0.15	0.58	0.11	1289.0	<0.001*
P_{50}-Diameter	1.80	0.56	2.15	0.32	1520.0	0.002*
IQR-Diameter	0.61	0.26	0.73	0.22	1569.0	0.005*

(MSC as the connectivity method in Alpha-2 frequency band, the rank-sum test was used to compare the difference as normality was not satisfied, * $P<0.05$)

Table S4-15: Univariate analysis of dynamic network characteristic

Item	P_{50}_PC	*IQR*_PC	P_{50}_PE	*IQR*_PE	*W*	*P*
Mean-Little world index	1.05	0.04	1.04	0.05	3013.0	<0.001*
Standard Deviation-Little world index	0.14	0.04	0.16	0.02	1507.0	0.002*
P_{50}-Little world index	1.05	0.04	1.04	0.04	2873.0	0.002*
IQR-Little world index	0.18	0.05	0.19	0.04	1797.0	0.072
Mean-Average vertex strength	2.60	0.27	2.85	0.70	1105.0	<0.001*
Standard Deviation-Average vertex strength	0.46	0.16	0.59	0.23	1055.0	<0.001*
P_{50}-Average vertex strength	2.55	0.25	2.78	0.69	1213.0	<0.001*
IQR-Average vertex strength	0.60	0.25	0.76	0.38	1325.0	<0.001*
Mean-Average path length	1.58	0.01	1.58	0.00	1852.0	0.121
Standard Deviation-Average path length	0.03	0.01	0.03	0.01	1225.0	<0.001*
P_{50}-Average path length	1.57	0.00	1.57	0.01	1596.5	0.003*
IQR-Average path length	0.02	0.01	0.03	0.01	1312.5	<0.001*
Mean-Transitivity	0.45	0.01	0.45	0.01	2654.0	0.039*
Standard Deviation-Transitivity	0.04	0.01	0.05	0.01	1215.0	<0.001*
P_{50}-Transitivity	0.45	0.01	0.45	0.01	2640.0	0.045*
IQR-Transitivity	0.06	0.01	0.06	0.02	1618.0	0.009*
Mean-Diameter	0.81	0.09	0.90	0.22	1104.0	<0.001*
Standard Deviation-Diameter	0.18	0.06	0.23	0.10	1055.0	<0.001*
P_{50}-Diameter	0.78	0.08	0.86	0.23	1197.0	<0.001*
IQR-Diameter	0.23	0.09	0.29	0.12	1250.0	<0.001*

(iCOH as the connectivity method in Alpha-2 frequency band, the rank-sum test was used to compare the difference as normality was not satisfied, * $P<0.05$)

Table S4-16: Univariate analysis of dynamic network characteristic

Item	P_{50}_PC	*IQR*_PC	P_{50}_PE	*IQR*_PE	*W*	*P*
Mean-Little world index	1.02	0.05	1.06	0.04	1297.0	<0.001*
Standard Deviation-Little world index	0.12	0.04	0.12	0.03	2127.0	0.757
P_{50}-Little world index	1.01	0.05	1.04	0.05	1292.0	<0.001*
IQR-Little world index	0.16	0.04	0.16	0.04	2212.0	0.944
Mean-Average vertex strength	5.60	0.19	5.93	0.33	440.0	<0.001*
Standard Deviation-Average vertex strength	0.29	0.08	0.30	0.15	1808.0	0.080
P_{50}-Average vertex strength	5.56	0.18	5.90	0.33	466.0	<0.001*
IQR-Average vertex strength	0.38	0.12	0.38	0.20	1906.0	0.191
Mean-Average path length	1.86	0.05	1.87	0.04	1720.0	0.032*
Standard Deviation-Average path length	0.11	0.05	0.12	0.04	2041.0	0.485
P_{50}-Average path length	1.85	0.04	1.86	0.04	1686.0	0.021*
IQR-Average path length	0.12	0.05	0.12	0.04	2095.0	0.650
Mean-Transitivity	0.57	0.05	0.59	0.05	1663.0	0.016*
Standard Deviation-Transitivity	0.07	0.02	0.07	0.02	1908.0	0.194
P_{50}-Transitivity	0.57	0.05	0.58	0.05	1707.5	0.028*
IQR-Transitivity	0.09	0.02	0.10	0.04	2047.0	0.503
Mean-Diameter	2.18	0.22	2.40	0.32	866.0	<0.001*
Standard Deviation-Diameter	0.43	0.13	0.49	0.17	1438.0	0.001*
P_{50}-Diameter	2.15	0.25	2.34	0.29	927.0	<0.001*
IQR-Diameter	0.55	0.17	0.61	0.18	1571.0	0.005*

(CORR as the connectivity method in Alpha-2 frequency band, the rank-sum test was used to compare the difference as normality was not satisfied, * $P<0.05$)

Table S4-17: Univariate analysis of dynamic network characteristic

Item	P_{50}_PC	IQR_PC	P_{50}_PE	IQR_PE	W	P
Mean-Little world index	1.14	0.04	1.16	0.05	1400.0	<0.001*
Standard Deviation-Little world index	0.18	0.02	0.19	0.02	1272.0	<0.001*
P_{50}-Little world index	1.13	0.03	1.15	0.05	1235.0	<0.001*
IQR-Little world index	0.22	0.04	0.25	0.05	1267.0	<0.001*
Mean-Average vertex strength	1.77	0.47	2.28	0.59	780.0	<0.001*
Standard Deviation-Average vertex strength	0.32	0.12	0.46	0.20	818.0	<0.001*
P_{50}-Average vertex strength	1.72	0.44	2.24	0.44	769.0	<0.001*
IQR-Average vertex strength	0.39	0.15	0.60	0.29	834.0	<0.001*
Mean-Average path length	1.64	0.01	1.64	0.01	1554.5	0.00*
Standard Deviation-Average path length	0.04	0.01	0.04	0.01	1380.0	<0.001*
P_{50}-Average path length	1.64	0.01	1.64	0.01	1678.5	0.015*
IQR-Average path length	0.05	0.01	0.05	0.02	1567.0	0.004*
Mean-Transitivity	0.44	0.01	0.45	0.01	1220.0	<0.001*
Standard Deviation-Transitivity	0.05	0.01	0.05	0.01	1310.0	<0.001*
P_{50}-Transitivity	0.44	0.01	0.45	0.01	1290.0	<0.001*
IQR-Transitivity	0.06	0.01	0.06	0.01	1630.5	0.011*
Mean-Diameter	0.65	0.18	0.84	0.23	752.0	<0.001*
Standard Deviation-Diameter	0.15	0.05	0.22	0.11	787.0	<0.001*
P_{50}-Diameter	0.63	0.17	0.82	0.23	773.0	<0.001*
IQR-Diameter	0.19	0.07	0.27	0.12	864.0	<0.001*

(PLI as the connectivity method in Beta frequency band, the rank-sum test was used to compare the difference as normality was not satisfied, * P<0.05)

Table S4-18: Univariate analysis of dynamic network characteristic

Item	P_{50}_PC	IQR_PC	P_{50}_PE	IQR_PE	W	P
Mean-Little world index	0.93	0.06	1.02	0.13	640.0	<0.001*
Standard Deviation-Little world index	0.11	0.01	0.14	0.03	480.0	<0.001*
P_{50}-Little world index	0.92	0.06	1.01	0.13	798.0	<0.001*
IQR-Little world index	0.14	0.03	0.17	0.04	986.0	<0.001*
Mean-Average vertex strength	4.17	0.20	4.64	0.68	730.0	<0.001*
Standard Deviation-Average vertex strength	0.15	0.05	0.38	0.22	187.0	<0.001*
P_{50}-Average vertex strength	4.16	0.22	4.56	0.61	812.0	<0.001*
IQR-Average vertex strength	0.19	0.06	0.40	0.26	273.0	<0.001*
Mean-Average path length	1.82	0.03	1.81	0.05	2376.0	0.418
Standard Deviation-Average path length	0.06	0.02	0.11	0.05	386.0	<0.001*
P_{50}-Average path length	1.81	0.02	1.82	0.05	2011.0	0.404
IQR-Average path length	0.07	0.02	0.11	0.06	711.0	<0.001*
Mean-Transitivity	0.47	0.04	0.54	0.07	595.0	<0.001*
Standard Deviation-Transitivity	0.06	0.01	0.08	0.02	516.0	<0.001*
P_{50}-Transitivity	0.47	0.04	0.54	0.07	732.5	<0.001*
IQR-Transitivity	0.07	0.02	0.10	0.03	808.0	<0.001*
Mean-Diameter	1.22	0.17	1.52	0.44	766.0	<0.001*
Standard Deviation-Diameter	0.20	0.05	0.34	0.14	444.0	<0.001*
P_{50}-Diameter	1.18	0.15	1.46	0.39	853.0	<0.001*
IQR-Diameter	0.26	0.07	0.41	0.19	602.0	<0.001*

(MSC as the connectivity method in Beta frequency band, the rank-sum test was used to compare the difference as normality was not satisfied, * P<0.05)

Table S4-19: Univariate analysis of dynamic network characteristic

Item	P_{50}_PC	IQR_PC	P_{50}_PE	IQR_PE	W	P
Mean-Little world index	1.07	0.02	1.07	0.05	1627.0	0.010*
Standard Deviation-Little world index	0.10	0.01	0.13	0.03	809.0	<0.001*
P_{50}-Little world index	1.06	0.02	1.07	0.07	1570.0	0.005*
IQR-Little world index	0.13	0.02	0.17	0.05	819.0	<0.001*
Mean-Average vertex strength	1.12	0.05	1.59	0.45	107.0	<0.001*
Standard Deviation-Average vertex strength	0.15	0.04	0.41	0.14	172.0	<0.001*
P_{50}-Average vertex strength	1.10	0.05	1.51	0.51	68.0	<0.001*
IQR-Average vertex strength	0.19	0.05	0.51	0.30	285.0	<0.001*
Mean-Average path length	1.57	0.00	1.58	0.01	1203.5	<0.001*
Standard Deviation-Average path length	0.02	0.00	0.03	0.01	581.0	<0.001*
P_{50}-Average path length	1.57	0.01	1.57	0.01	1531.0	0.001*
IQR-Average path length	0.02	0.01	0.03	0.01	823.5	<0.001*
Mean-Transitivity	0.46	0.01	0.46	0.01	1404.0	<0.001*
Standard Deviation-Transitivity	0.03	0.01	0.04	0.01	611.0	<0.001*
P_{50}-Transitivity	0.46	0.01	0.46	0.02	1277.0	<0.001*
IQR-Transitivity	0.05	0.01	0.06	0.01	745.0	<0.001*
Mean-Diameter	0.34	0.02	0.49	0.13	110.0	<0.001*
Standard Deviation-Diameter	0.06	0.01	0.14	0.06	212.0	<0.001*
P_{50}-Diameter	0.33	0.02	0.46	0.13	94.0	<0.001*
IQR-Diameter	0.07	0.02	0.18	0.11	362.0	<0.001*

(iCOH as the connectivity method in Beta frequency band, the rank-sum test was used to compare the difference as normality was not satisfied, * P<0.05)

Table S4-20: Univariate analysis of dynamic network characteristic

Item	P_{50}_PC	*IQR*_PC	P_{50}_PE	*IQR*_PE	*W*	*P*
Mean-Little world index	1.02	0.05	1.06	0.05	1157.0	<0.001*
Standard Deviation-Little world index	0.12	0.04	0.12	0.03	2081.0	0.605
P_{50}-Little world index	1.01	0.05	1.05	0.05	1218.0	<0.001*
IQR-Little world index	0.15	0.05	0.15	0.03	2046.0	0.500
Mean-Average vertex strength	5.54	0.20	5.88	0.32	433.0	<0.001*
Standard Deviation-Average vertex strength	0.29	0.09	0.30	0.16	1790.0	0.067
P_{50}-Average vertex strength	5.49	0.19	5.87	0.32	481.0	<0.001*
IQR-Average vertex strength	0.36	0.13	0.38	0.19	1861.0	0.131
Mean-Average path length	1.86	0.05	1.86	0.04	1813.0	0.084
Standard Deviation-Average path length	0.11	0.05	0.11	0.04	1990.0	0.353
P_{50}-Average path length	1.85	0.05	1.86	0.04	1839.0	0.107
IQR-Average path length	0.12	0.05	0.12	0.04	2026.5	0.445
Mean-Transitivity	0.57	0.04	0.59	0.05	1576.0	0.005*
Standard Deviation-Transitivity	0.07	0.02	0.07	0.02	1822.0	0.092
P_{50}-Transitivity	0.57	0.05	0.58	0.06	1622.5	0.010*
IQR-Transitivity	0.09	0.03	0.09	0.03	2118.5	0.728
Mean-Diameter	2.14	0.24	2.38	0.31	895.0	<0.001*
Standard Deviation-Diameter	0.42	0.11	0.49	0.16	1409.0	<0.001*
P_{50}-Diameter	2.09	0.25	2.29	0.30	922.0	<0.001*
IQR-Diameter	0.53	0.18	0.58	0.20	1687.0	0.022*

(CORR as the connectivity method in Beta frequency band, the rank-sum test was used to compare the difference as normality was not satisfied, * P<0.05)

Table S4-21: Univariate analysis of dynamic network characteristic

Item	P_{50}_PC	*IQR*_PC	P_{50}_PE	*IQR*_PE	*W*	*P*
Mean-Little world index	1.14	0.04	1.15	0.04	1707.0	0.039*
Standard Deviation-Little world index	0.18	0.03	0.19	0.03	1395.0	<0.001*
P_{50}-Little world index	1.13	0.05	1.14	0.05	1658.0	0.022*
IQR-Little world index	0.23	0.03	0.24	0.05	1601.0	0.011*
Mean-Average vertex strength	1.77	0.47	2.26	0.61	749.0	<0.001*
Standard Deviation-Average vertex strength	0.32	0.12	0.47	0.20	783.0	<0.001*
P_{50}-Average vertex strength	1.71	0.46	2.22	0.50	750.5	<0.001*
IQR-Average vertex strength	0.39	0.14	0.63	0.26	787.0	<0.001*
Mean-Average path length	1.64	0.01	1.64	0.01	1528.0	0.004*
Standard Deviation-Average path length	0.04	0.01	0.04	0.01	1317.0	<0.001*
P_{50}-Average path length	1.64	0.01	1.64	0.01	1688.0	0.025*
IQR-Average path length	0.05	0.01	0.05	0.01	1423.0	0.001*
Mean-Transitivity	0.44	0.01	0.45	0.01	1142.0	<0.001*
Standard Deviation-Transitivity	0.05	0.01	0.05	0.01	1294.0	<0.001*
P_{50}-Transitivity	0.44	0.01	0.45	0.01	1261.5	<0.001*
IQR-Transitivity	0.06	0.01	0.07	0.01	1393.0	<0.001*
Mean-Diameter	0.66	0.18	0.84	0.23	742.0	<0.001*
Standard Deviation-Diameter	0.15	0.06	0.22	0.11	869.0	<0.001*
P_{50}-Diameter	0.62	0.18	0.82	0.21	737.0	<0.001*
IQR-Diameter	0.19	0.08	0.26	0.12	850.0	<0.001*

(PLI as the connectivity method in full frequency band, the rank-sum test was used to compare the difference as normality was not satisfied, * $P<0.05$)

Table S4-22: Univariate analysis of dynamic network characteristic

Item	P_{50}_PC	IQR_PC	P_{50}_PE	IQR_PE	W	P
Mean-Little world index	0.94	0.06	0.99	0.08	693.0	<0.001*
Standard Deviation-Little world index	0.10	0.02	0.12	0.03	1076.0	<0.001*
P_{50}-Little world index	0.93	0.06	0.98	0.09	790.0	<0.001*
IQR-Little world index	0.13	0.03	0.15	0.04	1329.0	<0.001*
Mean-Average vertex strength	4.16	0.22	4.49	0.42	632.0	<0.001*
Standard Deviation-Average vertex strength	0.13	0.04	0.25	0.15	143.0	<0.001*
P_{50}-Average vertex strength	4.14	0.23	4.43	0.44	689.0	<0.001*
IQR-Average vertex strength	0.16	0.06	0.30	0.17	234.0	<0.001*
Mean-Average path length	1.82	0.04	1.82	0.04	2215.0	0.933
Standard Deviation-Average path length	0.06	0.03	0.09	0.04	1035.0	<0.001*
P_{50}-Average path length	1.81	0.04	1.82	0.03	2040.0	0.482
IQR-Average path length	0.07	0.03	0.10	0.04	1235.0	<0.001*
Mean-Transitivity	0.49	0.05	0.53	0.03	1021.0	<0.001*
Standard Deviation-Transitivity	0.05	0.01	0.06	0.02	961.0	<0.001*
P_{50}-Transitivity	0.48	0.05	0.52	0.04	1188.0	<0.001*
IQR-Transitivity	0.07	0.02	0.08	0.02	1303.0	<0.001*
Mean-Diameter	1.19	0.21	1.45	0.29	733.0	<0.001*
Standard Deviation-Diameter	0.20	0.07	0.28	0.11	527.0	<0.001*
P_{50}-Diameter	1.17	0.21	1.40	0.27	809.0	<0.001*
IQR-Diameter	0.24	0.08	0.33	0.10	760.0	<0.001*

(MSC as the connectivity method in full frequency band, the rank-sum test was used to compare the difference as normality was not satisfied, * $P<0.05$)

Table S4-23: Univariate analysis of dynamic network characteristic

Item	P_{50}_PC	*IQR*_PC	P_{50}_PE	*IQR*_PE	*W*	*P*
Mean-Little world index	1.07	0.02	1.09	0.05	1257.0	<0.001*
Standard Deviation-Little world index	0.11	0.02	0.12	0.02	928.0	<0.001*
P_{50}-Little world index	1.07	0.02	1.08	0.05	1255.0	<0.001*
IQR-Little world index	0.14	0.03	0.16	0.03	1073.0	<0.001*
Mean-Average vertex strength	0.73	0.03	1.06	0.26	114.0	<0.001*
Standard Deviation-Average vertex strength	0.10	0.02	0.26	0.11	226.0	<0.001*
P_{50}-Average vertex strength	0.71	0.04	1.00	0.22	106.0	<0.001*
IQR-Average vertex strength	0.13	0.03	0.36	0.16	259.0	<0.001*
Mean-Average path length	1.57	0.00	1.58	0.01	1348.5	<0.001*
Standard Deviation-Average path length	0.02	0.00	0.02	0.01	892.0	<0.001*
P_{50}-Average path length	1.57	0.01	1.57	0.01	1662.0	0.009*
IQR-Average path length	0.02	0.01	0.02	0.01	1088.5	<0.001*
Mean-Transitivity	0.46	0.01	0.47	0.01	1220.0	<0.001*
Standard Deviation-Transitivity	0.04	0.00	0.04	0.01	983.0	<0.001*
P_{50}-Transitivity	0.46	0.01	0.47	0.01	1321.5	<0.001*
IQR-Transitivity	0.05	0.01	0.05	0.01	1188.0	<0.001*
Mean-Diameter	0.22	0.01	0.32	0.09	123.0	<0.001*
Standard Deviation-Diameter	0.04	0.01	0.09	0.04	181.0	<0.001*
P_{50}-Diameter	0.22	0.01	0.30	0.08	108.0	<0.001*
IQR-Diameter	0.05	0.01	0.12	0.06	179.0	<0.001*

(iCOH as the connectivity method in full frequency band, the rank-sum test was used to compare the difference as normality was not satisfied, * P<0.05)

Table S4-24: Univariate analysis of dynamic network characteristic

Item	P_{50}_PC	*IQR*_PC	P_{50}_PE	*IQR*_PE	*W*	*P*
Mean-Little world index	1.02	0.05	1.05	0.05	1168.0	<0.001*
Standard Deviation-Little world index	0.12	0.04	0.12	0.03	2057.0	0.532
P_{50}-Little world index	1.01	0.05	1.04	0.05	1213.0	<0.001*
IQR-Little world index	0.15	0.05	0.15	0.04	2099.0	0.663
Mean-Average vertex strength	5.53	0.20	5.88	0.32	446.0	<0.001*
Standard Deviation-Average vertex strength	0.29	0.09	0.30	0.16	1795.0	0.071
P_{50}-Average vertex strength	5.48	0.19	5.87	0.31	488.0	<0.001*
IQR-Average vertex strength	0.36	0.12	0.38	0.20	1856.0	0.125
Mean-Average path length	1.86	0.05	1.86	0.04	1834.0	0.103
Standard Deviation-Average path length	0.11	0.05	0.11	0.04	2020.0	0.428
P_{50}-Average path length	1.85	0.05	1.86	0.04	1881.5	0.156
IQR-Average path length	0.12	0.05	0.12	0.04	2105.0	0.683
Mean-Transitivity	0.57	0.05	0.59	0.05	1589.0	0.006*
Standard Deviation-Transitivity	0.07	0.02	0.07	0.02	1824.0	0.093
P_{50}-Transitivity	0.57	0.05	0.58	0.06	1619.0	0.009*
IQR-Transitivity	0.09	0.03	0.09	0.03	2039.0	0.480
Mean-Diameter	2.13	0.23	2.35	0.31	909.0	<0.001*
Standard Deviation-Diameter	0.42	0.11	0.48	0.16	1454.0	0.001*
P_{50}-Diameter	2.08	0.24	2.28	0.29	919.0	<0.001*
IQR-Diameter	0.54	0.17	0.60	0.18	1629.0	0.011*

(CORR as the connectivity method in full frequency band, the rank-sum test was used to compare the difference as normality was not satisfied, * P<0.05)

Supplementary Part S5

The accuracy of machine learning classifier with significant features

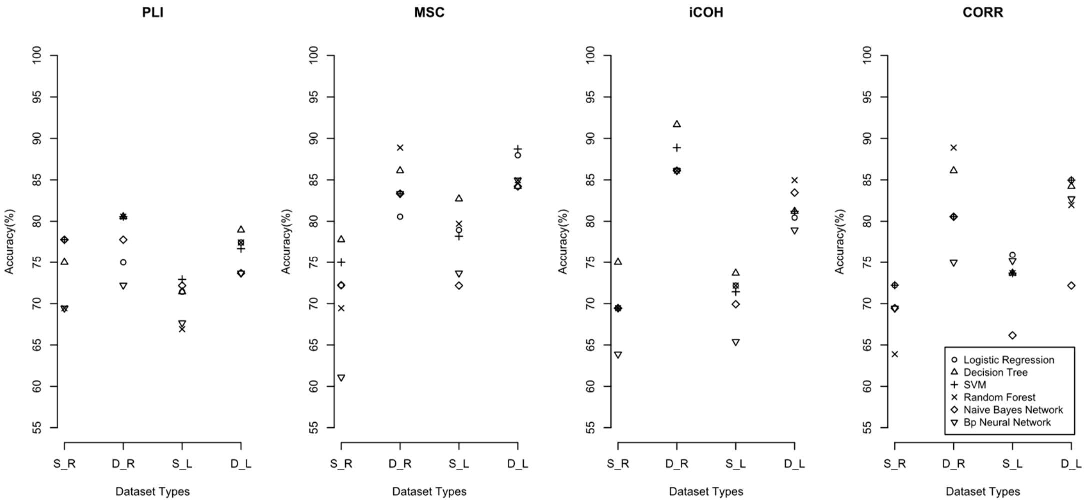

Figure S1. The accuracy of machine learning classifier with significant features under the Delta frequency band

(S: static network; D: dynamic network; L: split segment EEG signals dataset; R: original sequence EEG signals dataset; PLI: phase delay index; MSC: amplitude squared coherence; iCOH: coherence function Imaginary part; CORR: Pearson correlation coefficient)

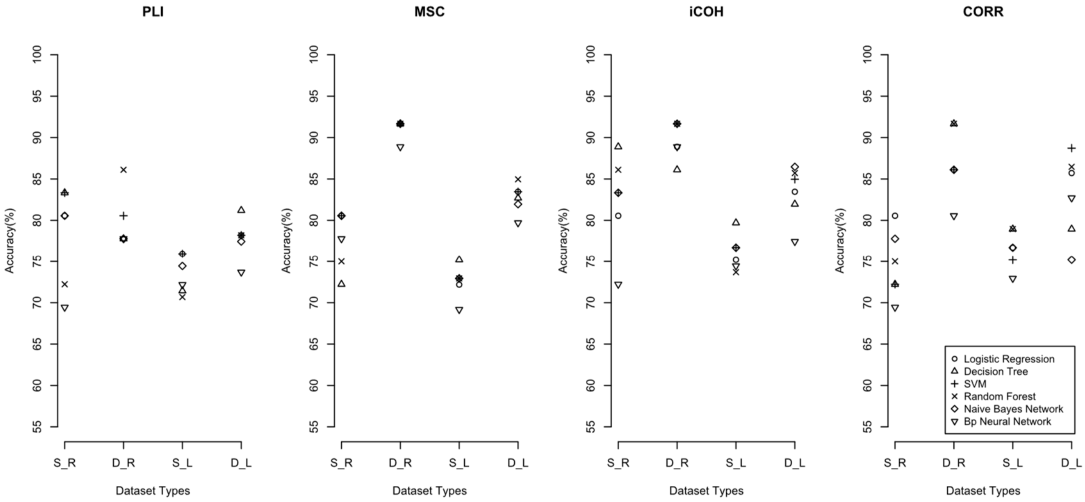

Figure S2. The accuracy of machine learning classifier with significant features under the Theta frequency band

(S: static network; D: dynamic network; L: split segment EEG signals dataset; R: original sequence EEG signals dataset; PLI: phase delay index; MSC: amplitude squared coherence; iCOH: coherence function Imaginary part; CORR: Pearson correlation coefficient)

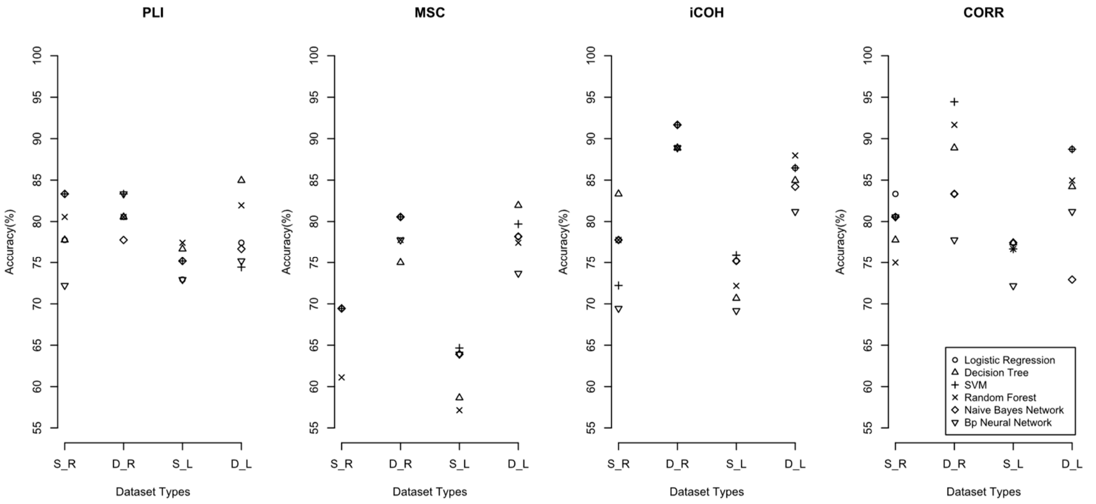

Figure S3. The accuracy of machine learning classifier with significant features under the Alpha-1 frequency band

(S: static network; D: dynamic network; L: split segment EEG signals dataset; R: original sequence EEG signals dataset; PLI: phase delay index; MSC: amplitude squared coherence; iCOH: coherence function Imaginary part; CORR: Pearson correlation coefficient)

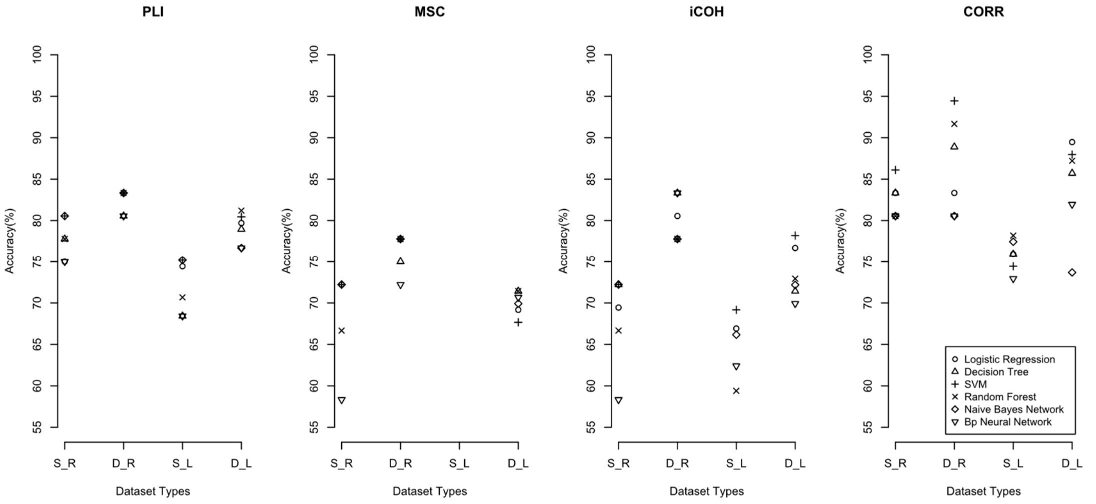

Figure S4. The accuracy of machine learning classifier with significant features under the Alpha-2 frequency band

(S: static network; D: dynamic network; L: split segment EEG signals dataset; R: original sequence EEG signals dataset; PLI: phase delay index; MSC: amplitude squared coherence; iCOH: coherence function Imaginary part; CORR: Pearson correlation coefficient)

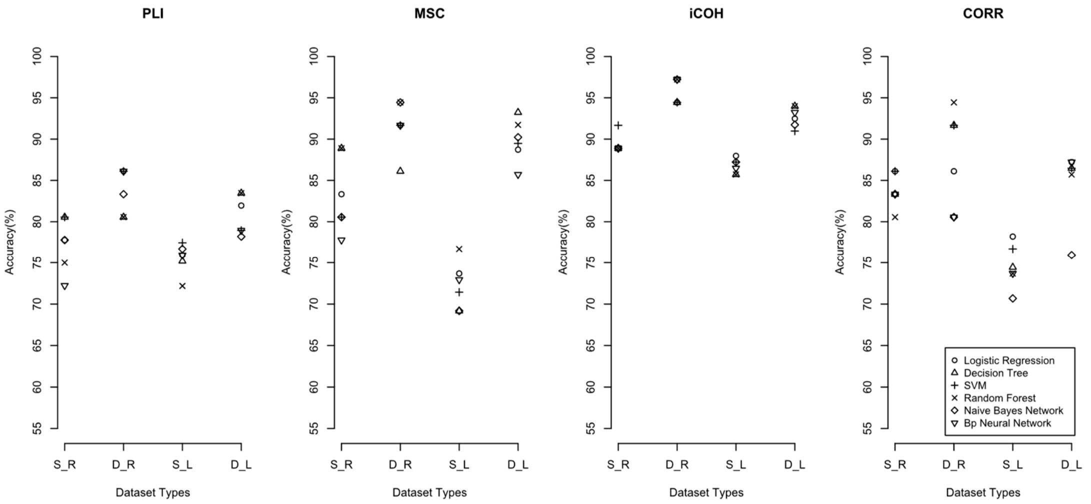

Figure S5. The accuracy of machine learning classifier with significant features under the Beta frequency band
(S: static network; D: dynamic network; L: split segment EEG signals dataset; R: original sequence EEG signals dataset; PLI: phase delay index; MSC: amplitude squared coherence; iCOH: coherence function Imaginary part; CORR: Pearson correlation coefficient)

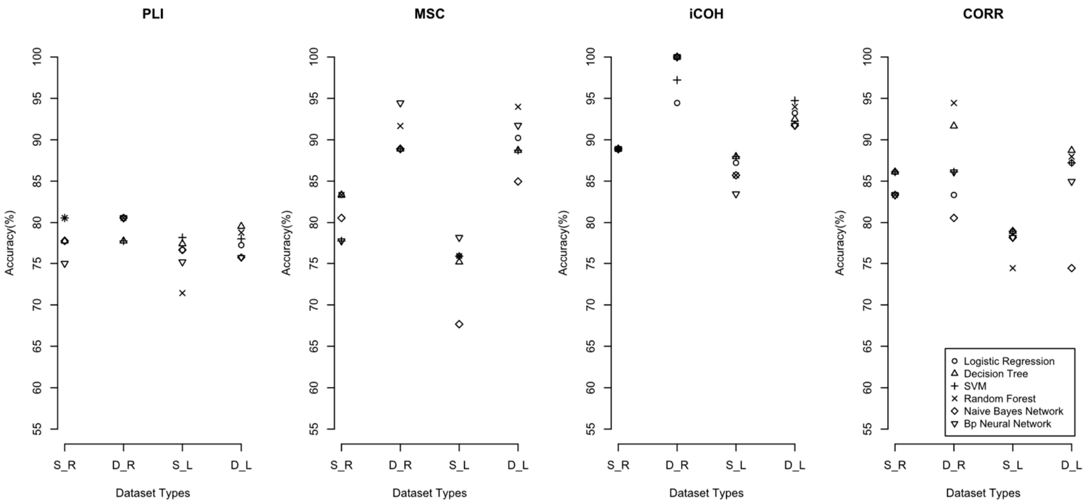

Figure S6. The accuracy of machine learning classifier with significant features under the Full frequency band
(S: static network; D: dynamic network; L: split segment EEG signals dataset; R: original sequence EEG signals dataset; PLI: phase delay index; MSC: amplitude squared coherence; iCOH: coherence function Imaginary part; CORR: Pearson correlation coefficient)
